# Bioinspired Passive Flow Routing to Mitigate Thrombosis in Prosthetic Heart Valves and Cardiovascular Devices

**DOI:** 10.1002/advs.76344

**Published:** 2026-07-03

**Authors:** Yevgeniy Kreinin, Mark Epshtein, Yahel Talmon, Tirosh Mekler, Itay Or, Mahli Raad, Gil Bolotin, Josué Sznitman, Netanel Korin

**Affiliations:** ^1^ Department of Biomedical Engineering Technion–Israel Institute of Technology Haifa Israel; ^2^ Department of Radiology New England Center for Stroke Research University of Massachusetts Medical School Worcester Massachusetts USA; ^3^ Department of Cardiac Surgery Rambam Health Care Campus Haifa Israel; ^4^ The Ruth Bruce Rappaport Faculty of Medicine Technion–Israel Institute of Technology Haifa Israel; ^5^ Fischell Department of Bioengineering University of Maryland, College Park MD United States

**Keywords:** biomimetic design, cardiovascular devices, computational fluid dynamics, hemodynamics, medical device engineering, passive flow modulation, thrombosis

## Abstract

Device‐associated thrombosis remains a critical problem in cardiovascular medicine, often requiring lifelong anticoagulation therapy that paradoxically introduces significant bleeding risks. Here, we present a bioinspired passive flow routing approach that mitigates thrombosis by altering local hemodynamics to reduce stagnation, a primary hemodynamic driver of clot formation. Inspired by flow‐reattachment mechanisms in avian wings and aeronautical slats, we integrate circumferential routing channels into mechanical heart valve housings to redirect a small fraction of forward flow into peri‐ring regions prone to stasis. Computational optimization identifies a configuration that eliminates near‐zero‐shear pockets and reduces exposure to low shear by several orders of magnitude, while maintaining comparable high‐shear exposure relative to control under physiologic conditions. In a fibrin clot deposition assay, the routed design exhibits reduced peri‐ring clot accumulation. In an ovine bypass model without anticoagulation, the routed valve demonstrated improved sinus washout across angiographic assessments and, unlike the control, explant examination after three months showed no macroscopic thrombus at the sewing‐ring interface. Preliminary computational extensions indicate that passive flow routing can alleviate stagnation in additional cardiovascular geometries. These findings establish bioinspired passive flow routing as a hemodynamic design strategy to mitigate thrombosis in cardiovascular devices by targeting the hemodynamic root cause of stasis.

## Graphical abstract

Bioinspired passive flow routing mitigates thrombosis in cardiovascular devices by redirecting a small fraction of forward flow into stagnation‐prone regions. Inspired by avian alula and aircraft slat mechanisms, strategically placed routing channels restore washout around mechanical heart valves, reducing low‐shear exposure by four orders of magnitude and preventing peri‐ring thrombosis in long‐term in vivo evaluation without anticoagulation. Preliminary computational studies indicate that this hemodynamic design principle may extend to other cardiovascular device geometries characterized by flow separation and stasis.

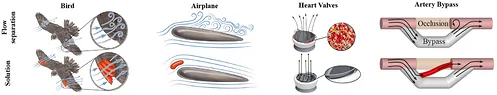

## 1 Introduction

Device‐associated thrombosis remains a significant challenge in cardiovascular medicine [1, 2, 3]. Blood‐contacting implants, including heart valves, vascular grafts, and stents, are inherently prone to thrombus formation and are therefore often managed with long‐term anticoagulation therapy despite its substantial bleeding risks [1, 4, 5]. Material‐based strategies, such as heparin bonding [6, 7, 8], phosphorylcholine coatings [9], and endothelialization [9, 10] approaches have improved hemocompatibility but have not eliminated thrombosis, in part because they do not address the underlying hemodynamic environment. Persistent flow stagnation and low shear [11, 12], rather than surface chemistry alone, remain central hemodynamic drivers of clot formation across cardiovascular devices [12].

This hemodynamic perspective aligns with Virchow's triad [11, 13], in which stasis, alongside hypercoagulability and endothelial injury, forms one of the three fundamental determinants of thrombogenesis. In regions where shear rates fall below ∼1 s^−^^1^, red blood cell aggregation, platelet adhesion, and accumulation of activated coagulation factors can proceed faster than they are transported away [14], enabling fibrin polymerization and stable clot formation [15, 16, 17]. Such low‐shear pockets are not incidental: they arise predictably from geometric flow separation in many different types of cardiovascular implants.

Flow separation and the resulting stagnation are universal fluid‐dynamic challenges [18] observed in both natural and engineered systems. As illustrated in Figure 1a, birds deploy an alula to generate reattachment vortices that prevent stall at high angles of attack [18, 19, 20], while aircraft use leading‐edge slats to energize the boundary layer and suppress separation [21, 22]. Conventional mechanical heart valves (MHVs), in which thrombosis is the primary cause of failure [23], exhibit a similar phenomenon: recirculation pockets routinely form around the sewing ring [23, 24], a region strongly associated with clot deposition in both clinical and experimental studies. Across valve platforms, thrombotic complications are described using different clinical and imaging terminology. In transcatheter and surgical bioprosthetic valves, thrombosis is often discussed in terms of its imaging manifestation, hypoattenuated leaflet thickening (HALT) [25], whereas in mechanical valves, thrombosis is more commonly recognized through functional or morphological abnormalities. Despite these differences in presentation, the underlying hemodynamic problem of flow stagnation remains central. Because these stagnation zones are well defined and hemodynamically reproducible, MHVs serve as an ideal proof‐of‐concept platform for evaluating whether targeted flow manipulation can selectively wash out peri‐ring and sinus stagnation‐prone regions, thereby suppressing stasis‐driven thrombogenesis. Although the present study is framed primarily through bioinspired flow‐separation and aircraft mechanisms, the broader concept of deliberately creating an auxiliary flow pathway also has a clinically relevant analog in the BASILICA procedure, in which intentional leaflet laceration is used to preserve downstream flow access during transcatheter valve intervention [26].

**FIGURE 1:**
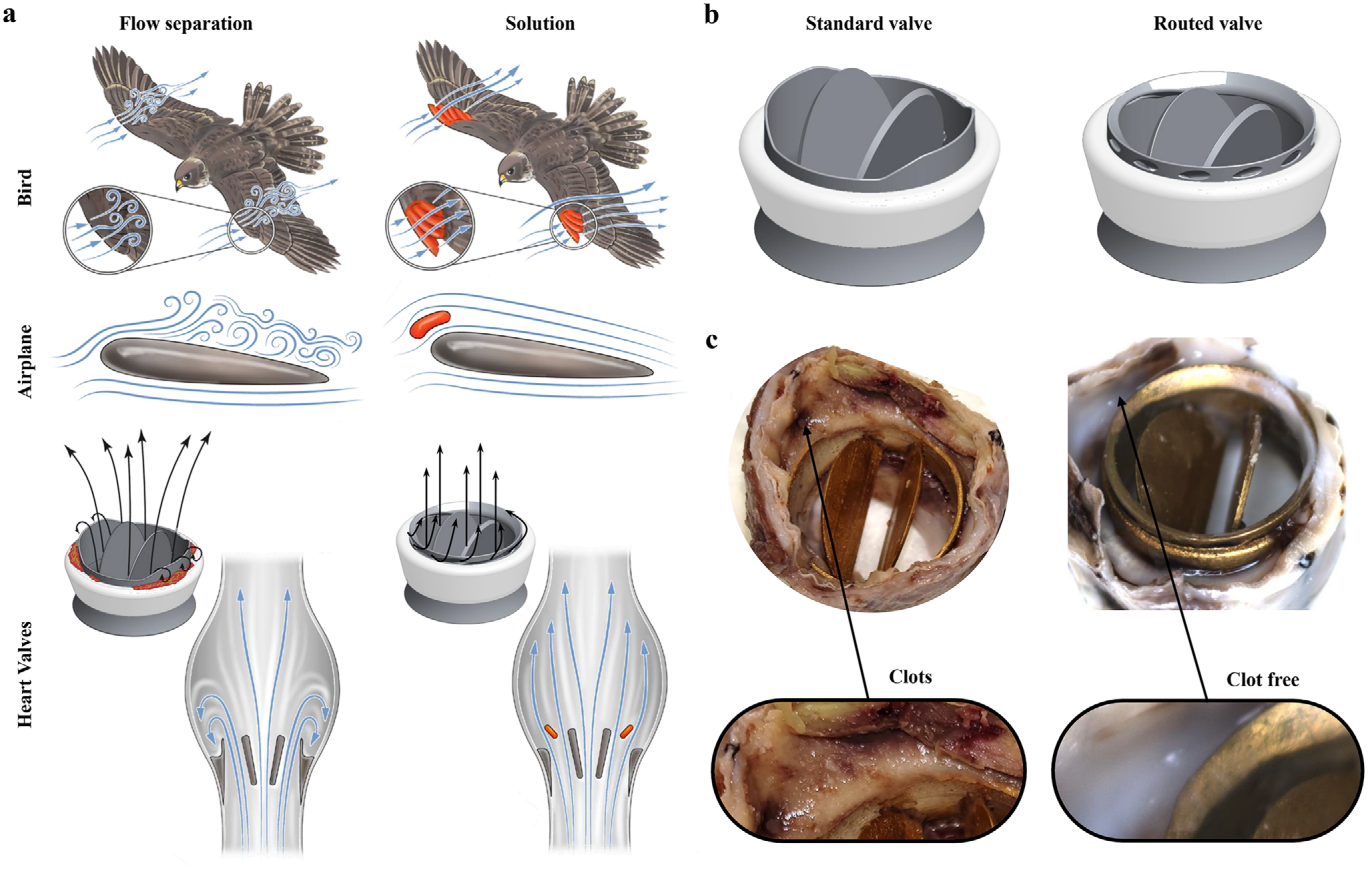
Bioinspired rationale and implementation of passive flow routing in mechanical heart valves. (a) Conceptual comparison illustrating flow separation in a bird wing, an aircraft slat, and a conventional bileaflet mechanical heart valve (MHV). Incoming flow separates over the wing surfaces and around the valve sewing ring, forming recirculation zones and peri‐ring stagnation pockets. Natural and engineered systems suppress flow separation by using flow‐reattachment mechanisms such as the avian alula or leading‐edge slats, which energize the boundary layer and suppress flow separation. (b) Computer‐aided design (CAD) renderings of the standard valve and the routed valve prototype, in which circumferential nozzles positioned above the sewing ring apply the same flow‐reattachment concept by directing a small portion of forward flow into low‐velocity peri‐ring regions. (c) Representative explanted control and routed valves showing macroscopic peri‐ring clot deposition in the control and a clean peri‐ring interface in the routed design.

Motivated by this bioinspired analogy, we introduce a passive flow‐routing strategy that integrates small circumferential channels into the valve housing to redirect a small fraction of the forward flow into peri‐ring regions prone to stagnation (Figure 1b). This approach contains no moving components and leverages intrinsic transvalvular pressure gradients to restore washout. A representative comparison (Figure 1c) shows the conceptual promise of this strategy: a standard valve exhibits a macroscopic clot at the sewing ring, whereas a routed configuration shows a clean peri‐ring interface, motivating deeper quantitative evaluation.

In this work, we establish passive flow routing as a hemodynamic design strategy for thrombosis mitigation, using MHVs as the principal demonstration case. We first computationally optimize routing geometries to eliminate low‐shear pockets and then validate the hemodynamic effects in vitro using a fibrin deposition assay. We further assess washout performance in an anticoagulation‐free ovine bypass model, as well as clot accumulation, and lastly, we extend the concept computationally to additional cardiovascular geometries. Together, these results demonstrate that targeted, bioinspired flow manipulation can suppress the hemodynamic conditions that initiate thrombosis in MHVs and may offer a broadly applicable strategy to improve the hemocompatibility of cardiovascular devices.

## 2 Results

### 2.1 Optimization of Flow Routing Reduces Stagnation Zones by Several Orders of Magnitude

Effective passive flow routing requires balancing circumferential coverage to eliminate stagnation zones while preserving central‐orifice hemodynamics. To examine how routing geometry influences washout near the sewing ring of mechanical heart valves, we performed computational fluid dynamics (CFD) simulations (see Methods, Figure S1 for detailed routing geometry, and Figure S2 for the longitudinal extraction plane used for the velocity contours) of a standard bileaflet valve (control) and routed variants that incorporated three (3R), six (6R), or fourteen (14R) circumferential nozzles, as shown in Figure 2a. Increasing nozzle count expands the angular coverage but reduces individual jet strength, thus requiring a systematic assessment of how nozzle distribution affects local hemodynamics. Additionally, each nozzle features a tangential exit orientation to generate coherent jets that spread circumferentially, improving washout and avoiding perpendicular impingement on the ring surface [27, 28], see Figure S1 for the detailed geometrical definitions of the 3R, 6R, and 14R routing configurations. The control valve exhibits extensive near‐zero‐velocity pockets (<0.02 m s^−^^1^) surrounding the sewing ring, whereas all routed designs redirect a portion of forward flow into these stagnant regions. Washout performance varied across configurations: the 3R geometry diverts 2.2% of flow and produces strong but localized jets; the 14R geometry diverts 2.5% with weaker jets and limited penetration; and the 6R configuration diverts 4.6% of flow while elevating peri‐ring velocities above 0.15 m s^−^^1^ across most of the circumference, yielding the most uniform washout pattern (Figure 2a); the common longitudinal XY extraction plane used for the velocity contours is shown in Figure S2.

**FIGURE 2:**
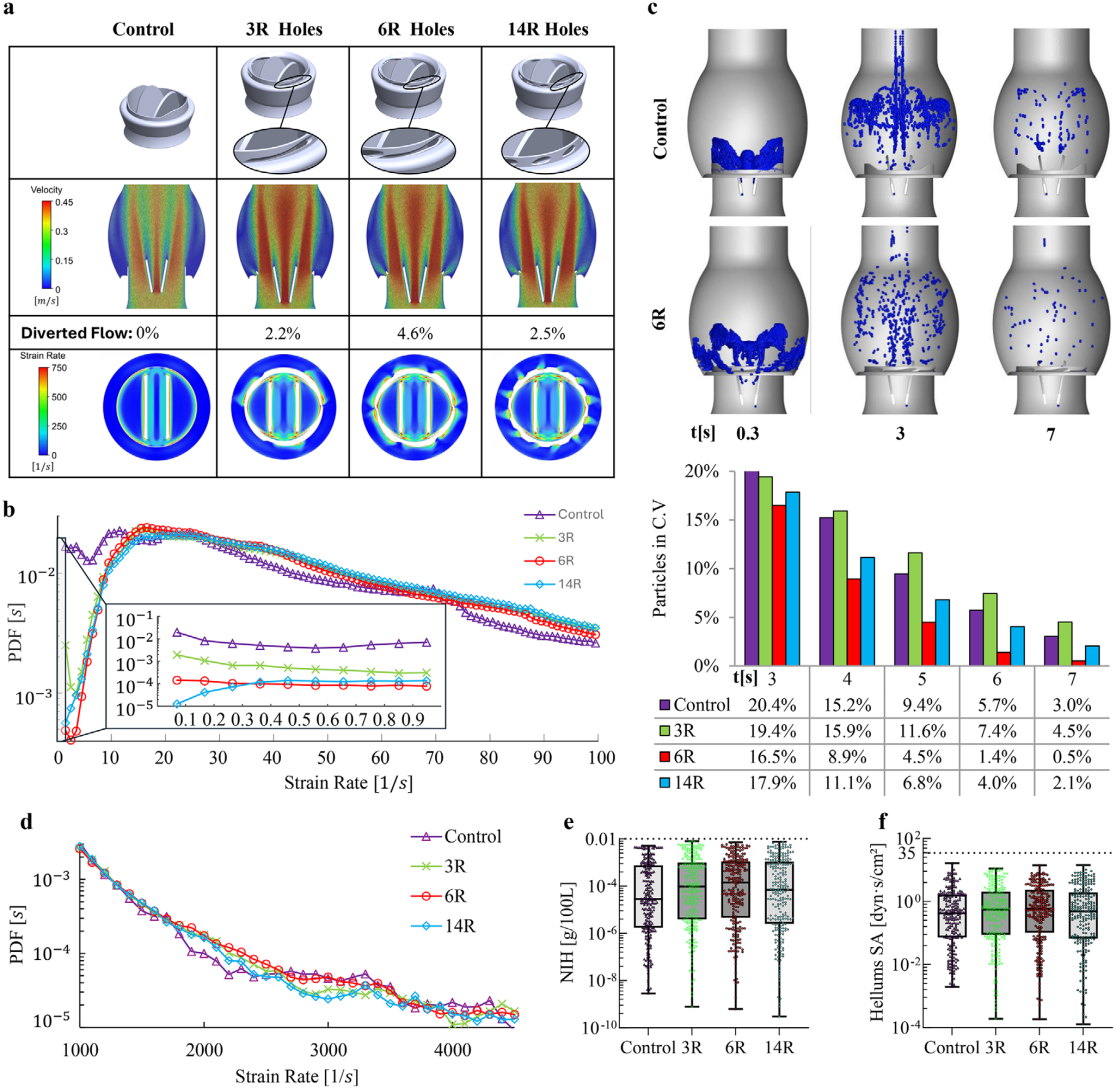
Comparative hemodynamics of control and routed mechanical heart valves. (a) Computational fluid dynamics (CFD) models and corresponding flow fields for the control valve and routed 3R, 6R, and 14R configurations. The top row shows the CAD models; the second row presents axial velocity maps at 5 L min^−^^1^, highlighting negligible peri‐ring velocity in the control and circumferential jets in routed valves. In the routed configurations, the insert that houses the flow‐routing channels also reduces the central outflow area of the primary forward‐flow path from 383 mm^2^ in the control valve to 305 mm^2^ in the routed valve. Accordingly, under the same imposed inlet flow rate, the remaining central forward flow becomes more spatially concentrated. The third row shows the fraction of forward flow diverted toward the sewing‐ring region. The bottom row displays strain‐rate maps above the sewing ring, where the control exhibits extensive low‐shear pockets and routed designs progressively reduce these zones, most prominently in the 6R configuration. (b) Probability density functions (PDFs) of strain rate sampled throughout the domain, showing decreased probability of low‐shear exposure (0.1–1 s^−^^1^) and increased probability of physiological shear (10–100 s^−^^1^) in routed designs relative to the control, with the 6R configuration exhibiting the lowest low‐shear probability. (c) Lagrangian particle tracking showing particle dispersion at 0.3, 3, and 7 s. The control valve demonstrates particle trapping in distinct recirculating structures near the sewing ring and sinus, while routed designs exhibit improved clearance, most notably 6R. Quantified particle residence between 3 and 7 s indicates retention of 3.0% for the control, 4.5% for 3R, 2.1% for 14R, and 0.5% for 6R, summarizing the relative washout efficiency across designs. (d) High strain rate (1000–5000 s^−^^1^) tail PDF sampled throughout the domain, plotted as PDF vs. strain rate, showing similar tail behavior across control, 3R, 6R, and 14R, indicating that routing does not increase the likelihood of extreme strain rate exposure under the tested condition. (e) Hemolysis metric distribution (NIH, g (100L)^−1^) computed from particle used in panel (c), displayed as boxplots with overlaid particle values on a log‐scaled NIH axis; all designs remain below the adopted NIH screening threshold of 0.01 g (100L)^−1^ at 5 L min^−^^1^. (f) Platelet stress accumulation distribution (Hellums SA, dyn s cm^−2^) computed from particle used in panel c, displayed as boxplots with overlaid particle values on a log‐scaled Hellums SA axis; all designs remain below the adopted activation criterion of 35 dyn s cm^−2^ at 5 L min^−^^1^.

Strain‐rate mapping showed broad low‐shear regions (<5 s^−^^1^) in the control valve, including persistent pockets below 1 s^−^^1^ associated with thrombogenesis. While the 3R and 14R designs reduced these pockets to varying degrees, the 6R configuration primarily decreased sub‐1 s^−^^1^ regions and increased exposure to physiological shear (10–100 s^−^^1^). To quantify these improvements, the probability density function (PDF) of the strain rate sampled across the fluid domain, which represents the relative likelihood that fluid elements experience a given shear value as they traverse the valve, was analyzed, providing a global measure of stagnation as shown in Figure 2b. Between the examined configurations, the 6R design shifts the distribution toward physiological values and exhibits the lowest probability density within the 0.1–1 s^−^^1^ stasis range. Importantly, the 6R flow‐routed valve shows an approximately four‐order‐of‐magnitude reduction in the low‐shear regions relative to the control, a shift that effectively removes the stagnation conditions known to promote thrombogenesis.

Lagrangian particle tracking (see Methods and Movie S1) was used to further characterize the ability of each routing configuration to clear recirculation zones. Shortly after release (0.3 s), all valves exhibited similar forward dispersion patterns; however, by 3 s, the control valve showed prominent particle trapping in distinct recirculating structures near the sewing ring and sinus. In contrast, the routed designs demonstrated progressively improved clearance, with the 6R configuration showing the most rapid reduction in near‐wall particle density. By 7 s, particle retention reached 3.0% for the control, 4.5% for 3R, and 2.1% for 14R, whereas the 6R valve retained only 0.5% of particles, as shown in Figure 2c. The sequential snapshots and quantitative retention curves in Figure 2c show that the 6R geometry reduces particle entrapment six‐fold relative to the control and by more than four‐fold relative to the next‐best routed design (14R). When considered alongside the velocity fields, peri‐ring strain‐rate maps, and global strain‐rate PDFs, these particle‐tracking results identify 6R as the configuration that most effectively suppresses stagnation while preserving organized forward flow.

To assess whether the routing channels reduce stasis without causing high‐shear complications, we conducted a hemocompatibility‐focused analysis centered on the high‐strain‐rate regime. As shown in Figure 2d, the high‐strain‐rate tail of the strain‐rate probability density function remained closely similar across the control, 3R, 6R, and 14R configurations over 1000 to 5000 s^−^^1^, indicating that the routed designs did not materially increase the probability of extreme strain‐rate exposure at a mean flow rate of 5 L min^−^^1^. To convert this observation into device‐relevant blood damage metrics, particle‐specific hemolysis was assessed with a Giersiepen‐type power‐law model, while platelet stress accumulation was analyzed using the Hellums criterion on the same Lagrangian trajectory used for residence time analysis [12, 13, 29, 30]. Consistent with the distributions shown in Figure 2e,f, all configurations remained below the adopted screening thresholds (*NIH*  =  0.01 *g* (100*L*)^−1^ for hemolysis, evaluated by the 95th percentile, and Hellums *SA*  =  35 *dyn* *s* *cm* ^−2^ for platelet activation, evaluated by the 99th percentile) [31, 32]. Importantly, within the routed valves, the 6R configuration showed the lowest platelet stress accumulation by the p99 criterion, while hemolysis remained minimal and similar across all routed designs and the control. Taken together, these results indicate that the substantial reduction in low‐shear exposure and particle retention achieved by the 6R design is not offset by increased high‐shear risk under the tested condition, identifying 6R as the configuration with the most favorable overall hemodynamic trade‐off at 5 L min^−^^1^. To assess peak systolic conditions, a high‐flow sensitivity analysis at 25 L min^−^^1^ was performed for the control and 6R configurations; under this extreme condition, hemolysis screening thresholds were exceeded for both designs, while platelet activation criteria (Hellums SA) remained below threshold, with detailed analysis provided in Figures S3 and S4.

### 2.2 Flow Routing Significantly Reduces Fibrin Clot Deposition in Vitro

To determine whether the hemodynamic improvements predicted by CFD suppress clot accumulation, we 3D‐printed models of the control valve and the 6R flow‐routed valve (see Figure S5) and evaluated fibrin deposition using a transport‐limited fibrin‐thrombin perfusion assay [33] (see Methods, Figure 3a). This closed‐loop pulsatile flow system recapitulates physiologic loading while promoting fibrin polymerization and clot accumulation primarily in regions with prolonged residence time. Following 60 min of perfusion, explanted valves exhibited distinct macroscopic differences (Figure 3b), where control valves exhibited continuous circumferential fibrin deposition along the sewing ring, whereas the 6R routed valves displayed only sparse, discontinuous deposits (Figure 3b). Angular distributions showed broad high‐deposition sectors in controls and narrow, low‐amplitude peaks in routed valves (Figure 3c). Quantitatively, peri‐ring fibrin coverage measured 2.0 ± 0.2% for controls and 0.3 ± 0.2% for 6R valves (*p* < 0.0001, *n* = 9), corresponding to a > 85% reduction (Figure 3d). Because fibrin formation in this system is transport‐limited, these results indicate that the enhanced washout produced by passive routing effectively suppresses fibrin clot accumulation [33, 34].

**FIGURE 3:**
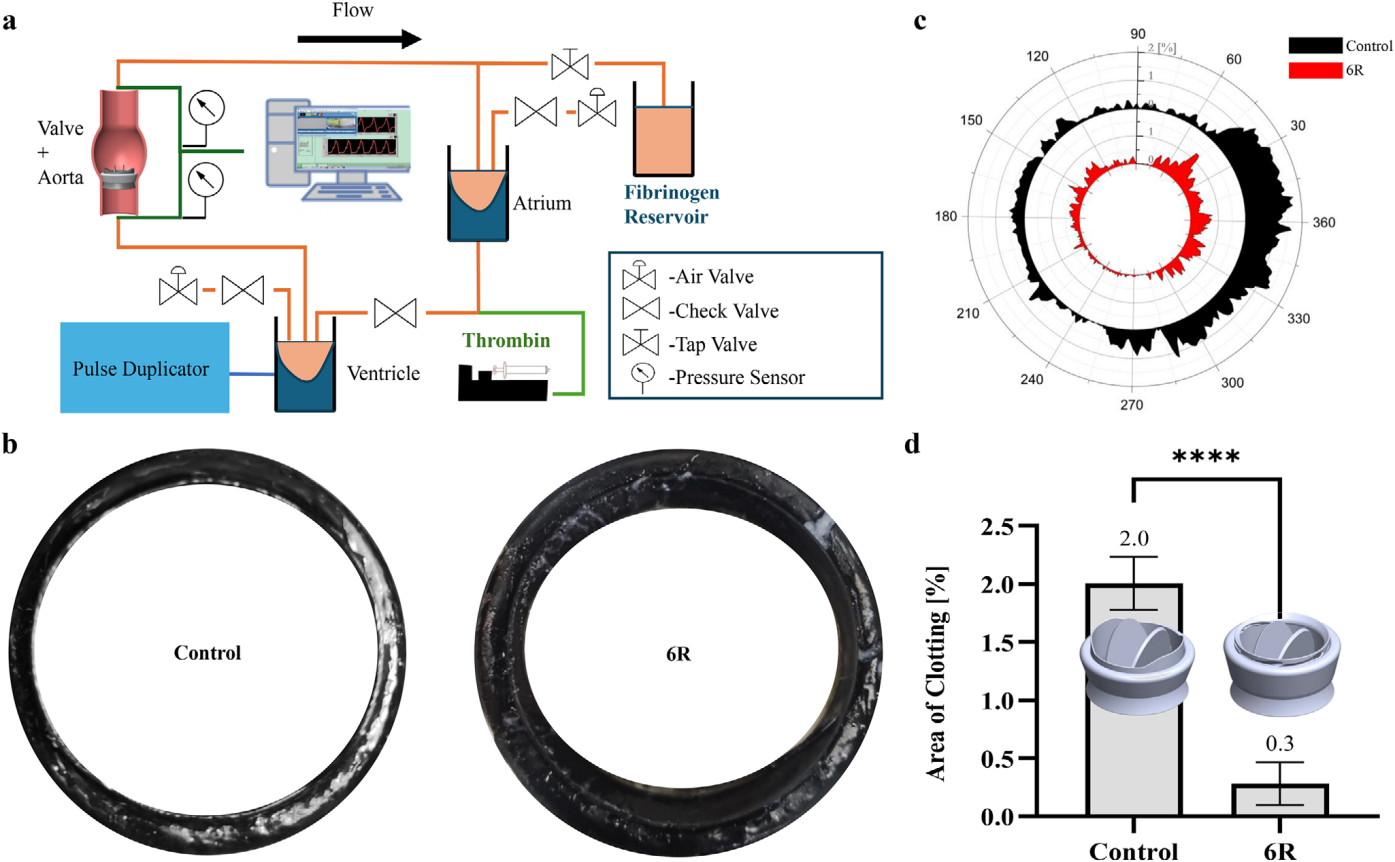
In vitro fibrin deposition in control and routed valves under transport‐limited clotting conditions. (a) Schematic of the closed‐loop pulsatile fibrin‐thrombin perfusion system used to reproduce physiologic aortic valve flow; a pulsatile pump drives flow through a ventricular chamber and transparent aortic root while fibrinogen circulates and thrombin is introduced upstream. (b) Representative sewing‐ring images after 60 min perfusion showing continuous fibrin deposition along the control valve sewing ring and sparse, discontinuous deposition in the routed 6R valve. (c) Angular distribution of clot coverage around the sewing ring for control and routed valves. (d) Quantification of total peri‐ring clot‐covered area demonstrates significantly reduced fibrin accumulation in the 6R configuration (*n* = 9, *p* < 0.0001).

### 2.3 Pilot In Vivo Study Demonstrates Improved Washout and No Thrombus

To examine passive flow routing under physiological loading in vivo, a routed 6R valve and a geometrically identical control valve were fabricated from titanium alloy [9, 35], coated with titanium nitride to enhance hemocompatibility (see Figure S6 for fabrication, TiN coating, and graft integration details), and implanted into a descending‐aorta bypass model in a sheep (Methods, Figure 4a,b). The 6R routed design and the conventional control valve were identical in material, dimensions, and surface treatment, differing only by the presence of six routing nozzles in the 6R variant (Supplementary Material). Each valve was mounted within a 24 mm diameter Valsalva graft designed to reproduce native aortic root geometry, and no anticoagulation (heparin, warfarin, or antiplatelet agents) was administered throughout the study to allow proper clot formation.

**FIGURE 4:**
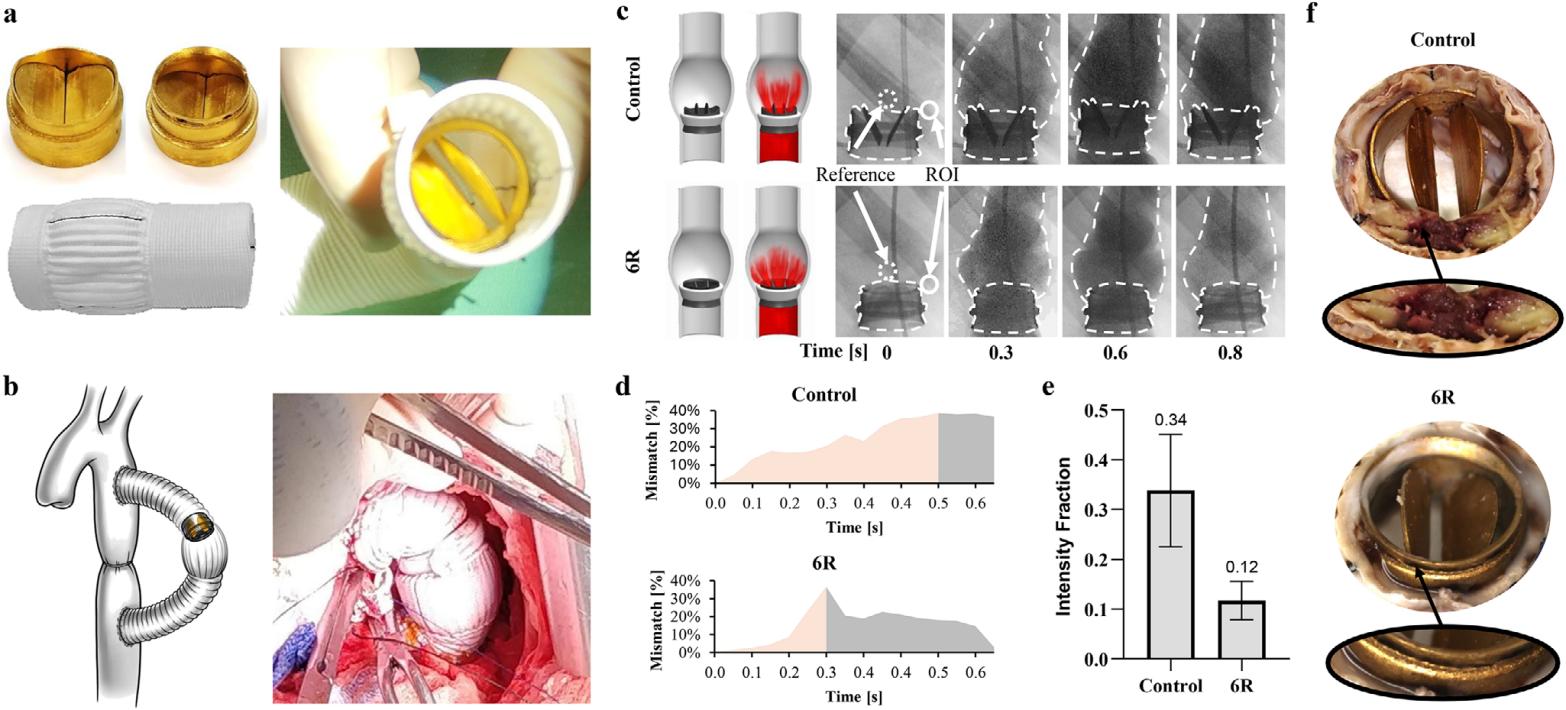
Pilot in vivo evaluation of washout and thrombus accumulation in a descending‐aorta bypass model. (a) Photographs of additively manufactured control and routed 6R mechanical valves, each fabricated from Ti6Al4V and coated with TiN, and the assembled valve‐Valsalva graft construct prior to implantation. (b) Schematic and intraoperative image of the descending aorta bypass configuration used to expose the test valves to systemic arterial flow. (c) Time‐resolved angiography showing contrast transport through control (top) and 6R (bottom) valves. A contrast bolus is injected upstream and tracked within a peri‐ring region of interest (solid outline circle) and a downstream reference region (dashed outline circle). Sequential frames illustrate delayed clearance and contrast persistence around the control valve, whereas the routed valve exhibits rapid, uniform washout. (d) Fluoroscopic video densitometry time‐intensity analysis of the temporal evolution of the normalized contrast‐intensity mismatch between the peri‐ring and reference regions, showing earlier peak filling and faster return toward baseline for the routed valve. (e) Summary of contrast‐retention values for each valve, reported as the mean ± SD across the three longitudinal measurement timepoints (1, 2, and 3 months). The retention metric is calculated as the average normalized intensity mismatch between the peri‐ring region of interest and the downstream reference region over the analyzed interval; lower values indicate more efficient sinus turnover. Because each valve represents a single biological sample, these values summarize temporal trends and are presented descriptively without inferential statistical testing. (f) Explanted valves after three months, showing circumferential peri‐ring thrombus on the control valve and no visible thrombus, with patent routing channels, on the routed valve.

Serial angiographic assessments performed at 1, 2, and 3 months demonstrated that the routed valve consistently exhibited rapid and homogeneous sinus washout (Figure 4c). In contrast, the control valve showed delayed clearance, with contrast persisting in peri‐ring regions for several cardiac cycles. Fluoroscopic video densitometry, implemented here as time‐intensity analysis of angiographic sequences, revealed lower normalized contrast‐mismatch values for the routed valve at each time point (Figure 4d). In particular, the retention metric, defined as the time‐average normalized intensity mismatch between the peri‐ring region of interest and the downstream reference region over the analyzed interval, was summarized across the three longitudinal measurements. The control valve exhibited a mean ± SD of 0.34 ± 0.11, whereas the routed valve measured 0.12 ± 0.04 (Figure 4e). Because each valve represents a single biological sample, these values characterize consistent temporal trends within each valve rather than statistical comparisons between groups.

At explant, following three months (> 5 million cardiac cycles), the control valve exhibited circumferential macroscopic peri‐ring thrombus, whereas the routed valve showed no visible thrombus and all routing channels remained open and did not become obstructed by clots (Figure 4f). While the in vivo study is a pilot in nature, the convergence of repeated, quantitatively distinct angiographic washout curves and the final thrombus‐free peri‐ring region supports the potential ability of routing channels to maintain washout and mitigate clot formation during chronic circulation without anticoagulation.

### 2.4 Application of the Passive Flow Routing Strategy to Other Cardiovascular Devices

Having established feasibility in mechanical valves, we next explored whether passive flow routing could be applied to other cardiovascular geometries characterized by flow separation and stagnation. To this end, we performed preliminary CFD analyses on two representative systems where low‐shear pockets are known to contribute to thrombosis: polymeric aortic valves and arterial bypass grafts (the corresponding idealized geometries are shown in Figure S7).

Polymeric tri‐leaflet valves, although designed to combine durability with improved hemodynamics, still exhibit regions of low shear behind leaflet backflow paths and near the sewing ring that have been associated with thrombus formation in prior studies [36, 37, 38]. In the control configuration, simulations reveal persistent low‐shear pockets adjacent to the sewing ring and behind commissural supports (Figure 5, top left). Introducing thin pressure‐responsive routing flaps redirects a fraction of systolic flow into these zones (Figure 5, top middle, and Supporting Information). Strain‐rate mapping showed that this modification elevated shear above the critical threshold of 1 s^−^^1^ in previously stagnant regions, and the corresponding strain‐rate PDF demonstrated reduced low‐shear probability (0.1–1 s^−^^1^) with increased representation of physiological shear (10–50 s^−^^1^) (Figure 5, top right).

**FIGURE 5:**
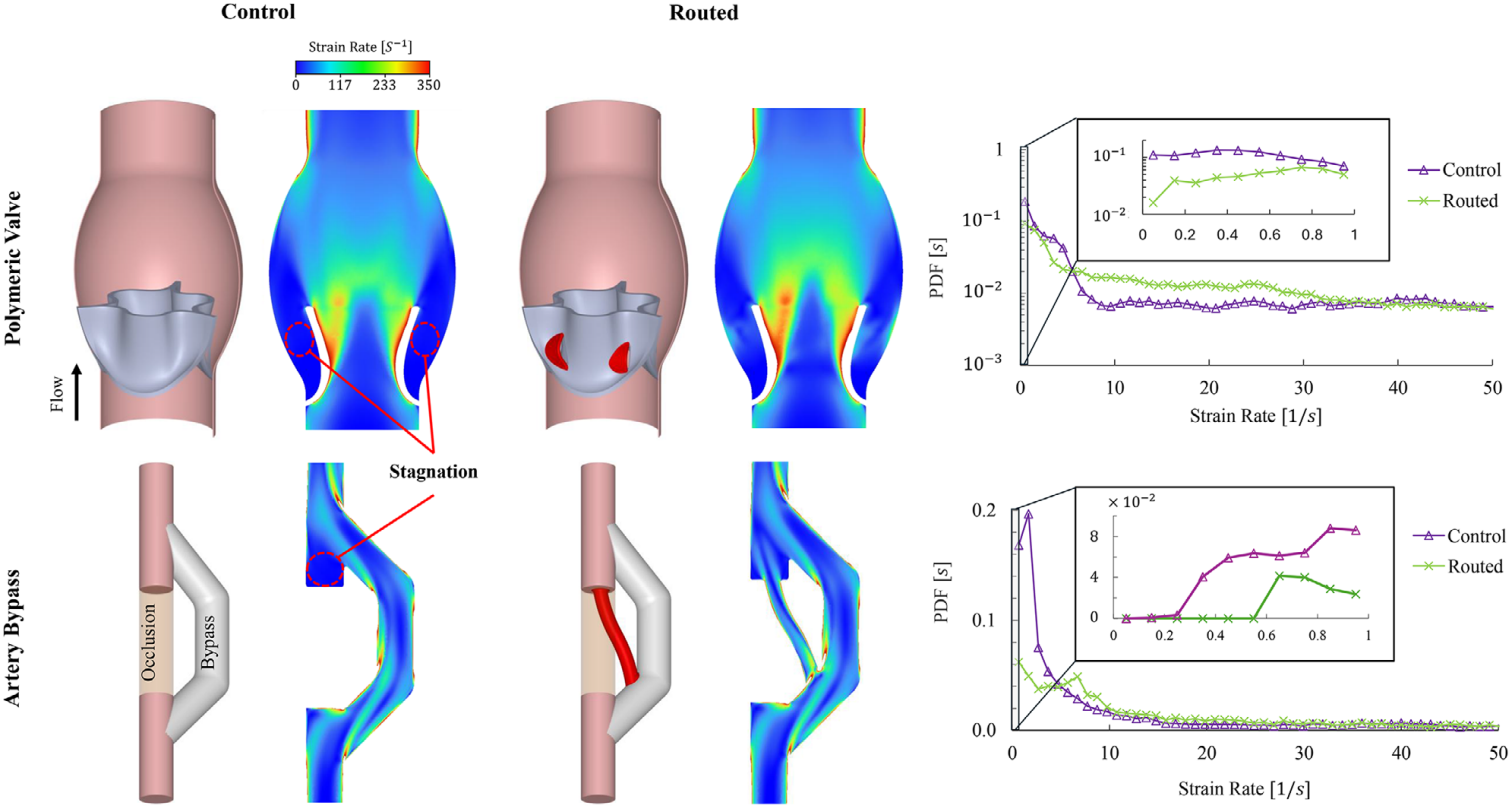
Preliminary CFD extension of passive flow routing to polymeric valves and arterial bypass grafts. (top row) Control and routed polymeric valve models with corresponding strain‐rate fields. In the control configuration, near‐zero–strain‐rate pockets appear adjacent to the sewing ring and along the leaflet backdrop (left), indicating local stasis. In the routed configuration, compliant flaps deflect a fraction of systolic flow into these regions (middle), elevating shear above the stagnation threshold and producing more continuous washout around the ring. (right) Probability density functions (PDFs) summarize this shift, showing reduced low‐shear (<1 s^−^^1^) exposure and increased representation of physiological shear (10–50 s^−^^1^). (bottom row) Control and routed arterial bypass graft models illustrating flow behavior around a fully occluded native artery. The control case exhibits a pronounced low‐shear pocket distal to the occlusion and at the anastomotic heel (left). Introducing a pressure‐equalizing conduit redirects flow into the stagnant segment and increases local shear (middle), as reflected in the corresponding strain‐rate maps and PDFs (right).

A similar stagnation‐driven thrombotic risk exists in arterial bypass grafts, where flow separation at the anastomotic heel and in the blind‐ended native artery promotes thrombosis and graft failure [39, 40]. In the fully occluded bypass configuration, control simulations exhibited a large stagnation pocket distal to the occlusion (Figure 5, bottom left). Adding a small pressure‐equalizing conduit can redirect flow into the stagnant region [39, 41] (Figure 5, bottom middle), restoring washout and increasing local shear. This modification again shifts the strain‐rate PDF away from low‐shear values associated with thrombus initiation (Figure 5, bottom right).

Together, these preliminary simulations indicate that passive flow routing can be adapted beyond bileaflet mechanical valves to additional cardiovascular geometries in which thrombosis‐promoting stasis arises from localized flow separation. In both the polymeric valve and bypass‐graft models, routing restored washout in stagnation‐prone compartments and shifted local strain‐rate distributions away from thrombogenic low‐shear ranges. These findings support the feasibility of extending the routing concept to additional device architectures, while emphasizing the need for application‐specific optimization and experimental validation [40, 41, 42, 43, 44, 45].

## 3 Discussion

This study establishes passive flow routing as a hemodynamic strategy to prevent device‐associated thrombosis by targeting flow stagnation at its source, rather than relying on biochemical surface modification. Across computational, in vitro, and in vivo evaluations, we find that directing approximately 5% of forward flow into stagnation‐prone regions substantially reduces pathological low‐shear pockets and improves washout conditions. This geometric intervention reduced macroscopic clot formation in a long‐term large‐animal study model without the use of anticoagulation, suggesting that correcting local hemodynamics is a feasible strategy for improving the safety of cardiovascular implants.

Mechanically, passive flow routing improves washout not only by flushing peri‐annular stagnation‐prone recesses, but also by reorganizing the primary valve outflow field. The routing insert redirects a small fraction of the forward flow into low‐washout regions while concentrating the remaining core stream, thereby contributing to the stronger outlet jet observed in the routed configuration. This effect should be interpreted as a redistribution of a fixed inflow rather than as an increase in total transvalvular flow. Thus, the hemodynamic benefit of passive routing arises from a coupled mechanism, local restoration of peri‐ring washout, together with global reorganization of the forward flow field.

Current clinical approaches to reducing device thrombosis primarily rely on systemic anticoagulation, which carries inherent bleeding risks [4, 5], or on material surface modifications. While surface treatments such as heparin bonding [7] or biomimetic coatings [42] address blood‐material interactions, they do not resolve the underlying issue of bulk flow stagnation. If a fluid domain remains stagnant, activated factors will eventually accumulate to concentrations that overcome the surface modification effects [10, 15, 43]. Furthermore, biological and physical coatings are subject to degradation and wear over the lifespan of a permanent implant [6, 9].

In contrast, passive flow routing creates a hydrodynamic barrier to thrombosis that functions independently of surface chemistry and can be synergetic to these material‐based studies. The valves used in our in vivo study were manufactured from standard titanium alloy with a generic titanium nitride coating. Nevertheless, the routed design showed negligible thrombosis accumulation, indicating that geometric control of local flow can provide a substantial antithrombotic benefit even in the absence of specialized surface engineering.

Passive flow routing should also be interpreted in the context of other geometry‐based efforts to improve mechanical valve hemocompatibility. Prior studies have shown that leaflet‐shape refinement can alter intraventricular flow organization and improve washout in bileaflet mechanical valves, while trileaflet mechanical valve architectures have been proposed as alternative strategies to reduce hinge‐related thrombogenicity and improve overall hemodynamics [27, 44, 45, 46, 47, 48]. Our approach differs in both target and mechanism. Rather than redesigning leaflet kinematics or replacing the bileaflet concept, passive routing introduces a dedicated washout pathway for stagnation‐prone peri‐ring recesses that may persist even when the primary transvalvular jet remains functional. Passive routing should therefore be viewed less as a competing alternative than as a complementary geometric layer that could, in principle, be integrated with leaflet optimization, alternative hinge designs, or trileaflet mechanical platforms.

Our results align with the mechanical foundation of Virchow's triad [11], specifically the role of stasis. The in vitro reduction of fibrin deposition by >85% in the routed valve, despite supraphysiological thrombin concentrations, validates the hypothesis that transport‐limited kinetics can be controlled via geometry [33, 49]. The in vivo survival of the 6R valve without anticoagulation highlights the clinical relevance of this approach. Current clinical management for mechanical valves mandates lifelong anticoagulation with associated bleeding risks [1, 5]. The ability to maintain thrombus‐free valves with reduced pharmacologic intervention suggests a potential pathway to reduce or eliminate this therapeutic burden for patients.

The preliminary computational extension of this work suggests that passive flow routing is not limited to mechanical heart valves, but may instead represent a broader hemodynamic design framework for cardiovascular devices in which thrombosis is driven by localized flow separation and washout failure. The polymeric‐valve and bypass‐graft simulations indicate that the central routing mechanism, namely identification of a stagnation‐prone compartment and redirection of pressure‐driven flow to restore turnover within it, remains effective across distinct materials, structural architectures, and anatomical settings. In polymeric valves, which seek to combine durability with improved hemocompatibility, routing may help address persistent sinus‐region stasis that is not eliminated by leaflet compliance alone [37, 38, 43, 50]. In bypass grafts, a related strategy may restore turnover in blind‐ended distal segments that are otherwise susceptible to prolonged stagnation, and thereby improve local hemodynamic conditions linked to long‐term graft performance [39, 40, 41, 51].

Beyond demonstrating feasibility in additional geometries, these extension studies also begin to define a broader design logic for passive flow routing. Across the device classes examined here, several common principles emerged. First, routing is most effective when it targets a reproducible stagnation compartment identified through velocity, strain‐rate, or residence‐time fields. Second, the routing feature should be positioned upstream of, or immediately adjacent to, that compartment so that the native pressure gradient drives flow into the stagnant zone. Third, the redirected flow should be distributed over sufficient circumferential or axial coverage to restore washout throughout the at‐risk region while keeping the diverted fraction small enough to preserve the primary device function. Fourth, the outlet geometry should favor attached sweeping flow rather than perpendicular impingement or the generation of secondary recirculation pockets. In valve applications, this was achieved through tangential circumferential streams or compliant flaps that washed peri‐annular or sinus recesses, whereas in bypass grafts it was achieved through a pressure‐equalizing conduit that restored turnover in an otherwise blind segment. In the bypass setting specifically, the present simulations were intended to evaluate washout recovery in the blind‐ended distal segment rather than to optimize anastomotic hemodynamics, and therefore should not be interpreted as addressing the risk of intimal hyperplasia at the graft junction [27, 36, 37, 39, 40, 41, 44, 45, 46, 47, 48]. Taken together, these observations suggest that passive flow routing should be viewed not simply as a valve‐specific geometric modification, but as a geometry‐dependent hemodynamic design strategy for suppressing thrombosis‐promoting stasis across a broader class of cardiovascular devices.

Our study has several limitations. First, the primary CFD analyses were performed under steady flow at 5 L min^−^^1^, which captures mean forward flow and provides a conservative framework for identifying stagnation regions, but does not resolve transient pulsatile acceleration and deceleration phases. To partly address peak‐flow conditions, we added a high‐flow sensitivity case at 25 L min^−^^1^ and report hemolysis and platelet stress‐accumulation distributions together with channel wall shear‐stress maps in Figures S3 and S4. Under a peak‐flow sensitivity condition (25 L min^−^^1^), hemolysis screening thresholds were exceeded for both control and routed configurations, whereas platelet stress accumulation remained below activation thresholds. This observation reflects an extreme instantaneous condition rather than a sustained physiological state. These findings highlight the need for further investigation under fully pulsatile physiological flow conditions, including experimental validation of hemolysis and platelet activation in device‐specific settings.

In addition, because the routed insert modifies the effective central outflow path, future studies should quantify the associated tradeoff between improved peri‐ring washout and possible changes in transvalvular pressure drop, effective orifice area, and energetic performance under pulsatile physiological loading. Second, the mechanical valve hinge region was not represented in the present CFD and in vitro sewing‐ring‐focused analyses. Clinically, thrombus often localizes to the hinge and hinge‐adjacent regions in bileaflet mechanical valves, and this distribution can vary across designs. The present findings should therefore be interpreted as demonstrating that passive routing can mitigate peri‐ring and sinus stasis, rather than as establishing a complete solution for all thrombosis mechanisms in bileaflet mechanical valves. Future work should incorporate hinge‐resolved geometries and assess whether the routing concept can be extended to hinge washout without introducing a high‐shear penalty. The fibrin‐thrombin assay isolates transport‐dependent clotting but does not include platelet or inflammatory components of thrombosis [33, 34]. In addition, the present in vivo assessment focused on local washout and macroscopic thrombus and did not include systemic biomarkers of hemolysis, nor did it quantify downstream microthromboembolic burden.

Furthermore, the in vivo assessment, while demonstrating consistent hemodynamic differences, was limited to a pilot comparison between one control and one 6R‐routed valve. Both implanted valves were custom titanium prototypes manufactured by direct metal laser sintering and coated with TiN; accordingly, the animal findings should be interpreted comparatively within this matched prototype platform rather than as direct evidence of equivalence to a commercial pyrolytic‐carbon control valve. Finally, the aortic‐root and bypass‐graft geometries used for the computational extension were intentionally idealized to isolate the effect of routing on stasis. Importantly, these geometries were not arbitrarily constructed, but were based on commonly used idealized configurations consistent with prior experimental and computational studies [40, 41, 51]. Future work should therefore evaluate anatomically refined and patient‐specific geometries, including smoother anastomotic transitions and joint optimization of blind‐end washout and anastomotic hemodynamics.

Taken together, these limitations do not alter the central implication of the present study. Across computational, in vitro, and pilot in vivo evaluations, the data support the view that thrombosis‐promoting flow stagnation can be mitigated through targeted geometric redistribution of flow, without an inherent high‐shear penalty under the screened operating conditions. More broadly, the significance of passive flow routing lies not only in the specific 6R configuration advanced here, but also in establishing a mechanically grounded framework for redesigning thrombosis‐prone cardiovascular devices to deliberately control local flow residence and washout.

## 4 Conclusions

This study demonstrates that passive flow routing, drawing inspiration from natural flow‐reattachment mechanisms such as the avian alula and aircraft slats [18, 19, 21, 22], offers a hemodynamics‐based strategy for thrombosis prevention in cardiovascular devices. By using small geometric adjustments to redirect a modest fraction of forward flow, this strategy restores physiological shear in stagnation‐prone regions, such as the sewing‐ring zone of mechanical heart valves, and thereby addresses a fundamental mechanical cause of thrombus initiation. The resulting normalization of shear complements rather than replaces existing biomaterial and pharmacologic approaches [6, 7, 9, 42] and provides a distinct design pathway for improving device hemocompatibility.

Preliminary computational extensions suggest that the same principle may be applicable to other cardiovascular devices in which geometry‐induced stasis contributes to thrombosis. Further studies are needed to validate passive routing under pulsatile flow, assess long‐term durability and performance in larger animal cohorts, and evaluate its adaptability across diverse device architectures.

Overall, passive flow routing represents a promising geometric design strategy for improving the safety of cardiovascular implants. If validated in expanded preclinical studies and eventually in clinical settings, this approach may help reduce dependence on systemic anticoagulation [4, 5] and contribute to the development of next‐generation thrombosis‐resistant cardiovascular devices.

## 5 Materials and Methods

### 5.1 Materials

For the experimental system preparation, perfusion, and cleaning, the following materials were used: Dulbecco's Phosphate Buffered Saline (D‐PBS), modified, without calcium chloride or magnesium chloride (Sigma‐Aldrich Inc.), was used as the main solution to which fibrinogen was added. Human Fibrinogen (Enzyme Research Laboratories Inc.) was added to the PBS (45 µg mL^−^^1^). Human α‐Thrombin (Enzyme Research Laboratories Inc., 3000 NIH unit mg^−^^1^) was dissolved in PBS (30 µg mL^−^^1^) and injected to convert fibrinogen to fibrin. To prime the system prior to clotting experiments, Bovine Serum Albumin (BSA) (Sigma‐Aldrich Inc.) was dissolved in PBS (1%) and perfused through the system. For washing and cleaning of the system, the following experiments were conducted: Sodium hypochlorite solution 120 g L^−^^1^ active chlorine was used. For the MHV and aorta model manufacturing, ABS‐like resin (3DM Advanced Materials Inc.) and Sylgard 184 22 Kg kit (PDMS) (Dow Corning) were used [33].

### 5.2 In Vitro Fibrin‐Thrombin Perfusion System

To emulate a realistic human aortic pulsatile flow, an experimental flow system was designed and built (see schematic in Figure 3a). The system contains a pulsatile pump (Harvard Apparatus 665) modified with a 115 cc hydraulic self‐produced piston. The pump was connected to the simulated ventricle chamber, separated by a flexible, sealed membrane made from a latex balloon. One side of the tank was connected via a tube to the pump, and the other to the aorta model with the MHV. The inlet and outlet of the aorta model were connected to pressure sensors (PendoTech Inc., PREPS‐N‐000 PressureMAT Single‐Use Sensor). The aortic outlet model was connected to the atrial chamber, which comprises a flexible membrane separating the circulating test fluid from a second fluid compartment. This second fluid compartment was not part of the clotting loop; rather, it functioned as a hydraulic compliance chamber that buffered cyclic pressure fluctuations and reproduced downstream compliance without mixing with the fibrinogen‐containing circulating solution. The atrium was connected to the ventricular chamber via a 3D‐printed one‐way valve. Two web cameras (Blaupunkt BP‐6310) were positioned to view the valve from above and from the side. The system flow conditions were defined to match physiological values and set at 60 beats per minute, with a stroke volume of 100 mL, resulting in an average flow rate of 6 L min^−^^1^, at a 30/70 systolic‐to‐diastolic ratio [33].

### 5.3 Formation of Fibrin Clots

Prior to the clotting experiments, to passivate the surfaces in the system, the fluid reservoir was filled with 500 mL of 1% BSA solution in D‐PBS, which was perfused through the system for 1 h. Following this step, the BSA solution was emptied, and the fluid reservoir was filled with a 500 mL human Fibrinogen solution (45 µg mL^−^^1^ in D‐PBS). This Fibrinogen solution is the main fluid that circulates in the system during experiments, whereas upon interaction with locally infused thrombin, the fibrinogen transforms to fibrin. The concentration of fibrinogen used in the system is much lower than its physiological level (20–40 mg mL^−^^1^) but still allows formation of stable fibrin clots. To produce the fibrin clots, a thrombin solution (30 µg mL^−^^1^, 90 U mL^−^^1^), which is approximately threefold higher than the threshold concentration (0.1 U mL^−^^1^) required for fibrin formation, is slowly injected into the system at an injection port located just before the valve (flow rate 1 mL h^−^^1^ using a Harvard Apparatus Elite 11 syringe pump) [33].

### 5.4 Aorta Model and 3D Printed MHVs

The MHV model was designed using SolidWorks software based on published On‐X manufacturer dimensions. The ONXAE‐29 valve model with an external sewing ring diameter of 34 mm was used [52]. The valve size, which is the largest produced by the company, was chosen because it is less sensitive to the resolution limitations of 3D‐printed valves. The valve was 3D‐printed (Elegoo Mars) using SLA technology and ABS‐like resin (see Supplementary Material). The valve's ring and leaflets were 3D‐printed separately and then assembled. Following the 3D printing and postprocessing, the valve leaflets were heated to 100°C, at which point the printed material becomes elastic, and the leaflets were inserted into the valve's ring. After the assembly, the valves were covered with a layer of transparent varnish to reduce moisture absorption. To improve the contrast of the fibrin clot and reduce its adhesion, the MHV sewing ring was painted black and then covered with transparent varnish [33].

### 5.5 Image Analysis

Image analysis was utilized to evaluate the clots accumulated on the valve. Initially, photographs of the valves were taken with time‐lapse imaging every minute during the experiment. To produce a quantification of the amount of clots deposited, the valve image at the end of each experiment was used. For this analysis, the region of interest, specifically the sewing ring on the valve, was isolated using ImageJ software. The grayscale threshold area of the clots was examined using the same software. Next, the pixel intensity of the valve in the grayscale area corresponding to the clots was evaluated and plotted using MATLAB software. Furthermore, time‐lapse images were used to observe the clots' accumulation as a function of time [33].

### 5.6 CFD Method

All the models were modeled by using SolidWorks (for the dimensions, see the appendix). All the models, meshes, and simulations were conducted in ANSYS Fluent 24 R2, solving for mass and momentum conservation (i.e., Navier‐Stokes equations) [23]. Blood flow was simulated under steady‐state conditions and assuming a Newtonian incompressible fluid. A fluid viscosity value of 3.5 mPa s and a density of 1060 kg m^−^^3^ were used to simulate blood. The inlet tube was significantly extended to allow for the flow to fully develop prior to entering the valve or the bypass, and at the inflow boundary, a normal inflow flow rate (5 L min^−^^1^) was specified as well as a constant pressure. Zero gradient conditions for the flow components were set at the outlet boundary, which was significantly extended to allow the outlet flow to recover. No‐slip and no‐flux conditions were specified for the velocity components on the rigid vessel walls, valves, and bypass surfaces [51, 53, 54]. For further specific simulation data, including case‐specific mesh cell counts and particle‐seeding parameters, see Table S1 and the Supplementary Methods. Hemocompatibility screening under high‐shear conditions was performed to address potential high‐shear risks introduced by routing channels. We computed two standard shear exposure screening metrics along Lagrangian particle trajectories: a Giersiepen‐type power‐law hemolysis index, which is an empirical hemolysis index in which stress‐based power law hemolysis estimates are empirical and best used for relative comparisons unless calibrated to device‐specific experimental data,  and a platelet stress accumulation (Hellums SA) criterion metric [13, 29, 30, 31, 55]. For each trajectory, the scalar shear stress was estimated from the local strain rate magnitude $\dot{\gamma }$ via $\tau =\mu \cdot \dot{\gamma }$ assuming Newtonian blood viscosity (μ  =  0.0035 *Pa* *s*) [27, 53, 54]. Hemolysis was estimated using a stress exposure power law model with constants *C* = 3.62 × 10^−7^, α = 2.416,  β = 0.785 and converted to an NIH like quantity in g (100 L)^−1^ using hemoglobin concentration *Hb*  =  140 *g* *L* ^−1^ and hematocrit *Hct*  =  0.40  [29, 30]. Platelet stress accumulation (Hellums SA) was computed as the trajectory integral of τ over time and reported in *dyn* *s* *cm* ^−2^. Screening criteria were NIH ≤ 0.01 g (100L)^−1^ (evaluated by the p95 rule) and Hellums *SA*  ≤  35 *dyn* *s* *cm* ^−2^ (evaluated by the p99 rule and by an activated‐fraction rule with an allowed fraction of 1%) [31, 32]. Hellums thresholding was interpreted as a model‐based screening criterion consistent with published use of the Hellums criterion in device thrombogenic risk analysis. In the primary 5 L min^−^^1^ analysis, all hemolysis and platelet activation metrics were computed using the same particle ensemble as the retention analysis to enable direct cross‐panel comparison. A high‐flow sensitivity case at 25 L min^−^^1^ was additionally evaluated for control and 6R and reported in Figures S3 and S4 to address peak flow conditions and channel shear [29]. Because both hemolysis and platelet activation models are empirical and sensitive to stress definition, turbulence closure, and coefficient selection, we interpret these results as comparative screening metrics under identical assumptions rather than as absolute clinical predictions. To support model credibility, mesh sensitivity is provided in Figure S8 [29, 30].

### 5.7 Animal Studies

In vivo studies were approved by the Technion Institutional Animal Care and Use Committee (protocol IL‐185‐11‐22) and performed in accordance with institutional guidelines and the Guide for the Care and Use of Laboratory Animals (National Research Council, eighth ed., 2011). Two adult female Awassi sheep (45–50 kg) underwent implantation of a control or 6R 23 mm valve model in a pilot feasibility study. Implantable valve models were scaled to 23 mm valves using SolidWorks software and fabricated by direct metal laser sintering (EOS M 290, EOS GmbH, Krailling, Germany) using Ti6Al4V alloy powder (EOS Titanium Ti64ELI, EOS GmbH, Krailling, Germany) in accordance with ASTM F2924, then integrated into a vascular graft conduit (TERUMO 730024ADP Vascutek Gelweave Valsalva Aortic Root Vascular Graft, 24 mm × 32 mm, Terumo Aortic, Inchinnan, Renfrewshire, United Kingdom). General anesthesia was initiated with ketamine hydrochloride (Ketaset 100 mg mL^−^^1^, Zoetis Manufacturing and Research Spain S.L., Girona, Spain; 10 mg kg^−^^1^, intravenous) and xylazine (Rompun 2%, Bayer Animal Health GmbH, Leverkusen, Germany; 0.1 mg kg^−^^1^, intravenous), followed by propofol (Propofol‐Lipuro 1%, B. Braun Melsungen AG, Melsungen, Germany; 5–7 mg kg^−^^1^, intravenous) to facilitate endotracheal intubation and mechanical ventilation; anesthesia was maintained with isoflurane (Isoflurane, USP, Piramal Critical Care, Inc., Bethlehem, PA, USA; 1.5%–2.5% in oxygen) and fentanyl (Fentanyl 50 microg mL^−^^1^ solution for injection, B. Braun Melsungen AG, Melsungen, Germany; 5–10 microg kg^−^^1^ h^−^^1^, intravenous infusion). A sterile left thoracotomy (fourth intercostal space) exposed the descending aorta, and local analgesia was provided with bupivacaine (Marcaine, distributed by Hospira, Inc., Lake Forest, IL, USA; 0.5%, 2 to 3 mL infiltrated at the incision site). Valve‐graft constructs were implanted by end‐to‐side anastomoses using polypropylene sutures (PROLENE 5‐0, Ethicon, Somerville, NJ, USA) in a continuous technique, and the native aorta between anastomoses was ligated to direct flow through the bypass. Postoperative analgesia consisted of tramadol (Trama Injection, Rafa Laboratories Ltd., Jerusalem, Israel; 100 mg, intramuscular, twice daily for 3 to 4 days) and tolfenamic acid (Tolfine 80 mg mL^−^^1^, Vetoquinol S.A., Lure, France; 4 mg kg^−^^1^, intramuscular, once daily starting 2 days preoperatively and continued for 5 days postoperatively); prophylactic antibiotics were administered for 10 days per institutional veterinary protocol (agent, manufacturer, and dosing recorded in the veterinary file). Perioperative thromboprophylaxis consisted of enoxaparin (Clexane 80 mg per 0.8 mL prefilled syringe, Sanofi, Reading, Berkshire, United Kingdom; 80 mg, subcutaneous, twice daily for 3 days). Clopidogrel was administered during a defined perioperative period (Plavix 75 mg, Sanofi, Paris, France), and all experimental evaluations were initiated only after completion of clopidogrel dosing, with no additional long‐term anticoagulation administered during the evaluation window.

At 1, 2, and 3 months, fluoroscopic angiography was performed under anesthesia. Radio‐opaque contrast (Visipaque 320 mg I mL^−^^1^, GE Healthcare, Chicago, IL, USA; 20 mL bolus) was power‐injected (10 mL s^−^^1^) while high‐speed fluoroscopy (15 fps) recorded sequences. Fluoroscopic video densitometry time‐intensity analysis used ImageJ (NIH, Bethesda, MD, USA). Mean grayscale intensity was measured over time in a peri‐ring region of interest, *I_ROI_* (*t*), and in a downstream reference region, *I_ref_* (*t*). The instantaneous mismatch was defined at each time point as the relative intensity difference between the two regions, *M*(*t*)  =  [*I_ROI_* (*t*) − *I_ref_* (*t*)]/*I_ref_* (*t*). The retention metric reported in Figure 4e was calculated as the time‐average of *M*(*t*) over the analysis window spanning peak filling through return toward baseline. The peri‐ring region was selected to interrogate the stagnation‐prone sinus compartment adjacent to the valve, whereas the downstream reference region served as an internal control for bulk bolus transport through the bypass.

At 3 months, animals were euthanized with pentobarbital (100 mg kg^−^^1^ IV under deep anesthesia). Constructs were explanted, flushed with heparinized saline, and photographed. Samples were fixed in 10% neutral buffered formalin.

### 5.8 Statistical Analysis

A t‐test was performed to evaluate statistical significance, where *p* < 0.05 denotes statistical significance. All error bars are depicted as the Standard Deviation (SD). In vitro fibrin data were compared via unpaired two‐tailed t‐tests. GraphPad Prism 9.0 (GraphPad Software, San Diego, CA, USA) was used for statistical analyses.

### 5.9 Ethics Approval and Patient Consent Statements

All procedures involving animals were conducted at the Preclinical Research Authority of the Technion Israel Institute of Technology and were reviewed and approved by the institutional Animal Ethics Committee (ethics approval IL‐185‐11‐22) and performed in accordance with institutional guidelines and the Guide for the Care and Use of Laboratory Animals (National Research Council, eighth ed., 2011). No human participants or identifiable human data were involved. Human fibrinogen and human α‐thrombin were commercially sourced reagents.

## Author Contributions

Conceptualization: Y.K., M.E. and N.K. Methodology: Y.K., M.E. and N.K. Investigation: Y.K., M.E., T.M., Y.T., I.O., M.R., G.B., J.S. and N.K. Resources: I.O., M.R., G.B., J.S. and N.K. Data curation: Y.K., Y.T. and N.K. Validation: Y.K., T.M., Y.T., I.O., M.R., G.B., J.S. and N.K. Formal analysis: Y.K. and N.K. Project administration: Y.K. and N.K. Visualization: Y.K., J.S. and N.K. Software: Y.K. and Y.T. Funding acquisition: N.K. Supervision: N.K. Writing – original draft: Y.K. and N.K. Writing – review and editing: all authors reviewed and approved the manuscript.

## Funding

This work was supported by the European Research Council (ERC) under the European Union's Horizon Europe Research and Innovation Program, ERC Proof of Concept Grant (StreamlineValve project, grant agreement #101123456), and the Israel Innovation Authority (Kamin grant #72268).

## Conflicts of Interest

Y.K., M.E., and N.K. are inventors on patent applications related to flow routing prosthetic heart valve designs described in this work (US20230255753A1 and EP4231967A1). All other authors declare that they have no competing interests.

## Supporting information




**Supporting File**: advs76344‐sup‐0001‐SuppMat.pdf.

**Supporting File**: advs76344‐sup‐0002‐Movie_V01.mp4.

## Data Availability

The data that support the findings of this study are available from the corresponding author upon reasonable request.
