# Supplementary material for: Bioinspired Passive Flow Routing to Mitigate Thrombosis in Prosthetic Heart Valves and Cardiovascular Devices
Source: Adv Sci (Weinh). 2026 Jul 3:e76344. Online ahead of print. doi: 10.1002/advs.76344 (PMC13334602; doi:10.1002/advs.76344)
Supplement: Supplementary file 1 — Supporting File: advs76344‐sup‐0001‐SuppMat.pdf. [file ADVS-9999-e76344-s002.pdf]

Supplementary Material For:

Bioinspired Passive Flow Routing to Mitigate Thrombosis in Prosthetic Heart Valves and Cardiovascular Devices

*Yevgeniy Kreinin[1],Mark Epshtein[1,2], Yahel Talmon[1], Tirosh Mekler[1], Itay Or[3], Mahli Raad[3], Gil Bolotin[3,4], Josué Sznitman[1], Netanel Korin[1] **

[1] Department of Biomedical Engineering, Technion - Israel Institute of Technology, 3200003 Haifa, Israel

[2]Department of Radiology, New England Center for Stroke Research, University of Massachusetts Medical School, Worcester, MA 01655, USA.

[3] Department of Cardiac Surgery, Rambam Health Care Campus, 3109601 Haifa, Israel

[4] The Ruth Bruce Rappaport Faculty of Medicine, Technion - Israel Institute of Technology, 3525433 Haifa, Israel

[*]Corresponding authors: Netanel Korin (E-mail: korin@bm.technion.ac.il)

Supplementary material includes:

Supplementary material text

Figure S1-S8

Movie V1

Table S1

Supplementary Methods: Computational Fluid Dynamics (CFD)

Solver Settings and Governing Equations

CFD simulations were performed to quantify how passive flow routing modulates stagnation and washout in the aortic sinus and peri-ring regions for multiple device geometries. The study analyzed a control mechanical heart valve (MHV). It routed mechanical variants (3R, 6R, 14R), polymeric tri-leaflet valves (control and routed), and arterial bypass grafts (control and routed via a pressure-equalizing conduit).

All geometries were solved in ANSYS Fluent using a pressure-based, three-dimensional, steady-state formulation. Turbulence was modeled using the standard k-epsilon model with standard wall functions. The working fluid was defined as blood with a density of 1060 kg m^{-3} and a dynamic viscosity of 0.0035 kg m^{-1} s^{-1}. Numerically, pressure-velocity coupling employed the SIMPLE algorithm. Momentum was discretized using a second-order upwind scheme, pressure was handled with second-order discretization, and turbulence parameters (k and ϵ) were discretized using a first-order upwind scheme. Convergence was enforced using residual targets of 10^{-6} for all solved equations, with additional solution monitors requiring inlet and outlet mass flow stability to within 10^{-6}. Initialization was performed using hybrid initialization.

Boundary Conditions and Mesh Generation

At the inlet, a mass flow condition of 0.08833 kg s^{-1} was imposed (equivalent to 5 L min^{-1}), and the outlet was defined as a pressure outlet. All walls were modeled as stationary with no-slip boundary conditions. To reduce boundary-driven artifacts and promote flow development and outlet recovery, the inlet and outlet domains were extended by 100 mm upstream and downstream of the device, respectively. Gravity was enabled with a magnitude of 9.81 m s^{-2} in the counterstream direction.

For the routed mechanical-valve configurations, the channel geometry was parameterized by outlet number, outlet width, outlet height, tangential deflection angle, and radial penetration angle. In the 3R and 6R designs, each outlet was 1.5 mm high and 11.8 mm wide, with a tangential deflection angle of 7.4° and a radial penetration angle of 44.1°. In the 14R design, each outlet was 2.2 mm high and 3.6 mm wide, with a tangential deflection angle of 24.2° and a radial penetration angle

of 39.4°. Across all designs, the internal diameter and sewing-ring curvature were preserved so that routing geometry remained the principal design variable. These dimensions are shown schematically in **Figure S1** and are stated here explicitly because small changes in outlet orientation and coverage strongly influence the resulting washout pattern and hemodynamic performance.

The computational domain was discretized using a polyhedral mesh. Total cell counts for each case are reported in **Table S1**. Mesh independence was verified to ensure that strain rate and velocity profiles were insensitive to further refinement.

Interpretation of velocity contours and effective central outflow area

In the routed valve configurations, the added insert served a dual role: it housed the circumferential flow-routing channels and also guided part of the forward flow into these channels. As a consequence, the effective central outflow area of the primary forward-flow path was reduced from 383 mm^2 in the control valve to 305 mm^2 in the routed valve. Therefore, for the same imposed inlet flow rate, the remaining central flow became more spatially concentrated, producing higher local axial velocities at the valve exit. For this reason, the velocity contours shown in **Figure 2a** should be interpreted as local field distributions extracted on an identical longitudinal plane, rather than as direct surrogates for total transvalvular flow rate.

High flow sensitivity and channel shear

To reflect peak systolic flow and ensure that nozzle-like routing features do not introduce a hemolysis risk, we evaluated an extreme forward flow condition (25 L min^{-1}) for both the control and 6R designs. Under this condition, the NIH hemolysis screening metric exceeded the 0.01 g $(100\ L)^{-1}$ threshold for both designs, whereas platelet stress accumulation (Hellums SA) remained below the activation criterion (35 dyn s cm^{-2}, p99). These findings indicate that threshold exceedance reflects a general high-flow sensitivity of the valve system rather than a routing-specific effect. Supplementary Figure S3 presents strain-rate tail distributions and particle-level exposure maps (NIH vs Hellums SA), while Supplementary Figure S4 provides spatial mapping of wall shear stress within the routing channels. These analyses serve as worst-case sensitivity screening and motivate future device-specific in vitro hemolysis testing under clinically relevant peak flow conditions.

Post-Processing and Lagrangian Particle Tracking

Post-processing focused on strain rate statistics and residence time metrics. A strain rate probability density function (PDF) was computed by extracting strain rate values across the entire analysis volume (defined as the full aortic sinus and valve flow domain) to provide a global measure of low-shear exposure. The PDF was generated using volume-weighted sampling and reported over physiologically relevant ranges: 0.1 to 1 s^{-1} for stasis and 10 to 100 s^{-1} for physiological shear.

To visualize washout and quantify residence time, inert Lagrangian particle tracking was performed. Particles were seeded through the valvular inlet region and tracked through the domain. This method was used to generate the comparative washout visualization for the control and 6R valves (see **Movie V1**), including snapshots at 0.3 s, 3 s, and 7 s. Additionally, a quantitative residence time analysis was performed by extracting residence time values over the complete control volume (aortic sinus and valve domain) and aggregating these values to generate the retention curves reported in **Fig. 2C**. Particle properties and specific seeding details for both the residence time analysis and visualization videos are provided in **Table S1**.

Mesh generation and numerical settings

To verify that the resolved flow field is not dominated by discretization error, we performed a mesh convergence assessment using three meshes (coarse, medium, fine) with nominal sizes of approximately 2M, 6M, and 11M cells, respectively. For a representative simulation condition in a 6R valve configuration at a flow rate of 5 L min^{-1}, velocity magnitude profiles were sampled along radial probe lines at six downstream stations (y = 5, 10, 15, 20, 25, 30 mm) and extracted in two orthogonal orientations (x-directed and z-directed), as shown in Supplementary Figure S8. Taking the fine mesh as reference, the medium mesh reproduced the velocity profiles with a mean relative L2 error of 2.33% across all 12 profiles (maximum 3.45%), corresponding to a mean RMS difference of approximately 0.025 m s^{-1} (maximum absolute deviation approximately 0.106 m s^{-1}). The coarse mesh showed larger differences (mean relative L2 error 4.61%, maximum 9.22%), with the largest deviations occurring near steep gradients associated with jet boundaries and separation regions. Based on this analysis, the fine mesh was selected as the production mesh for the main simulations.

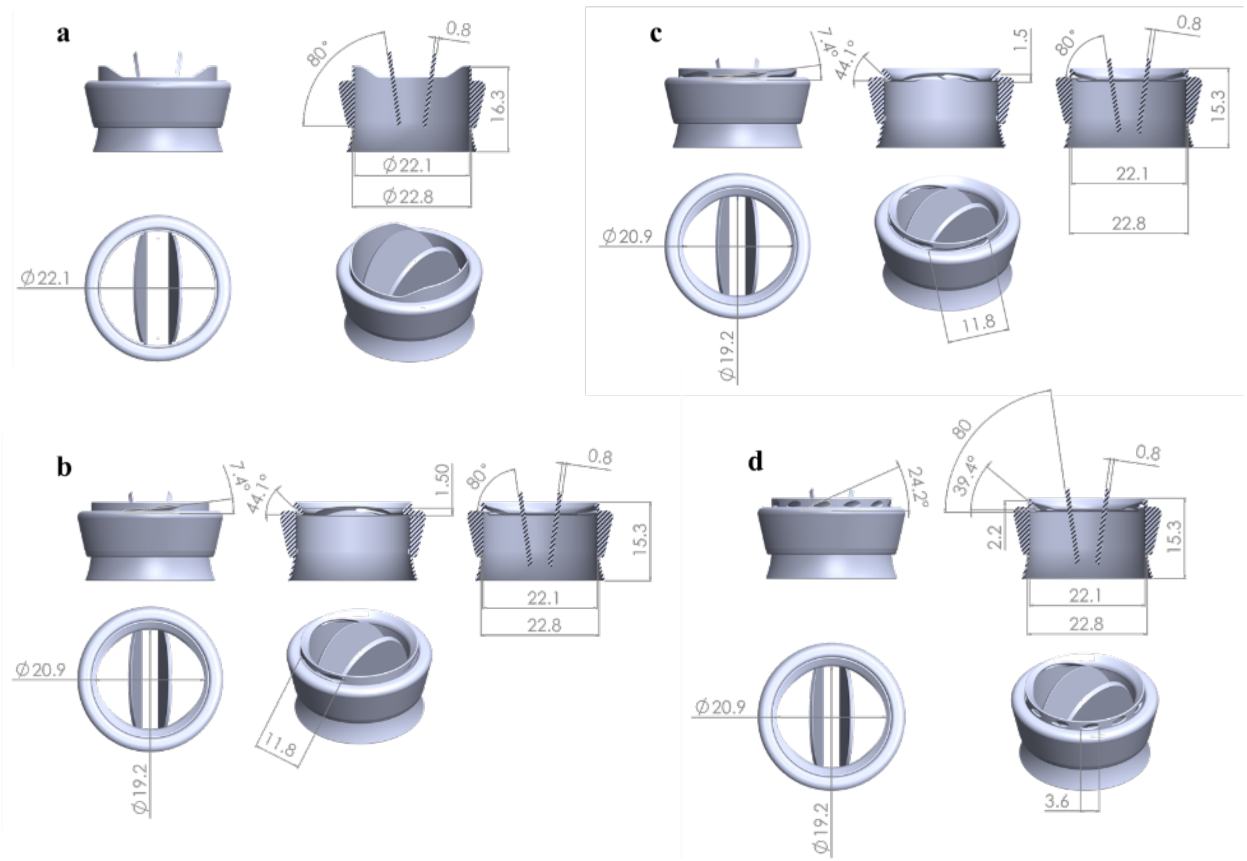

Figure S1. Engineering design and dimensions of mechanical heart valve housings. (a) Technical drawings of the mechanical control heart valve housing showing the sewing ring and housing dimensions, with a leaflet opening angle of 80°. (b) Design specifications for the 3R (three-channel) routed configuration. Cross-sectional view of the valve housing, showing the orientation of the channel exit. The channel exit is oriented tangentially to the sewing ring circumference, with a deflection angle of 7.4° and penetration (radial) angle of 44.1°, channel height of 1.5 mm, and width of 11.8 mm. (c) Design specifications for the 6R (six-channel) routed configuration—cross-sectional view of the valve housing showing the channel exit orientation. The channel exit is oriented tangentially to the sewing ring circumference, with a deflection angle of 7.4° and penetration (radial) angle of 44.1°, channel height of 1.5 mm, and width of 11.8 mm. (d) Design specifications for the 14R (fourteen-channel) routed configuration—cross-sectional view of the valve housing showing the channel exit orientation. The channel exit is oriented tangentially to the sewing ring circumference, with a deflection angle of 24.2°, a radial (penetration) angle of 39.4°, a channel height of 2.2 mm, and a width of 3.6 mm. All dimensions

are in millimeters; angles are in degrees. The internal diameter (ID) and sewing ring curvature are maintained consistently across all variants to isolate the effect of the routing channels.

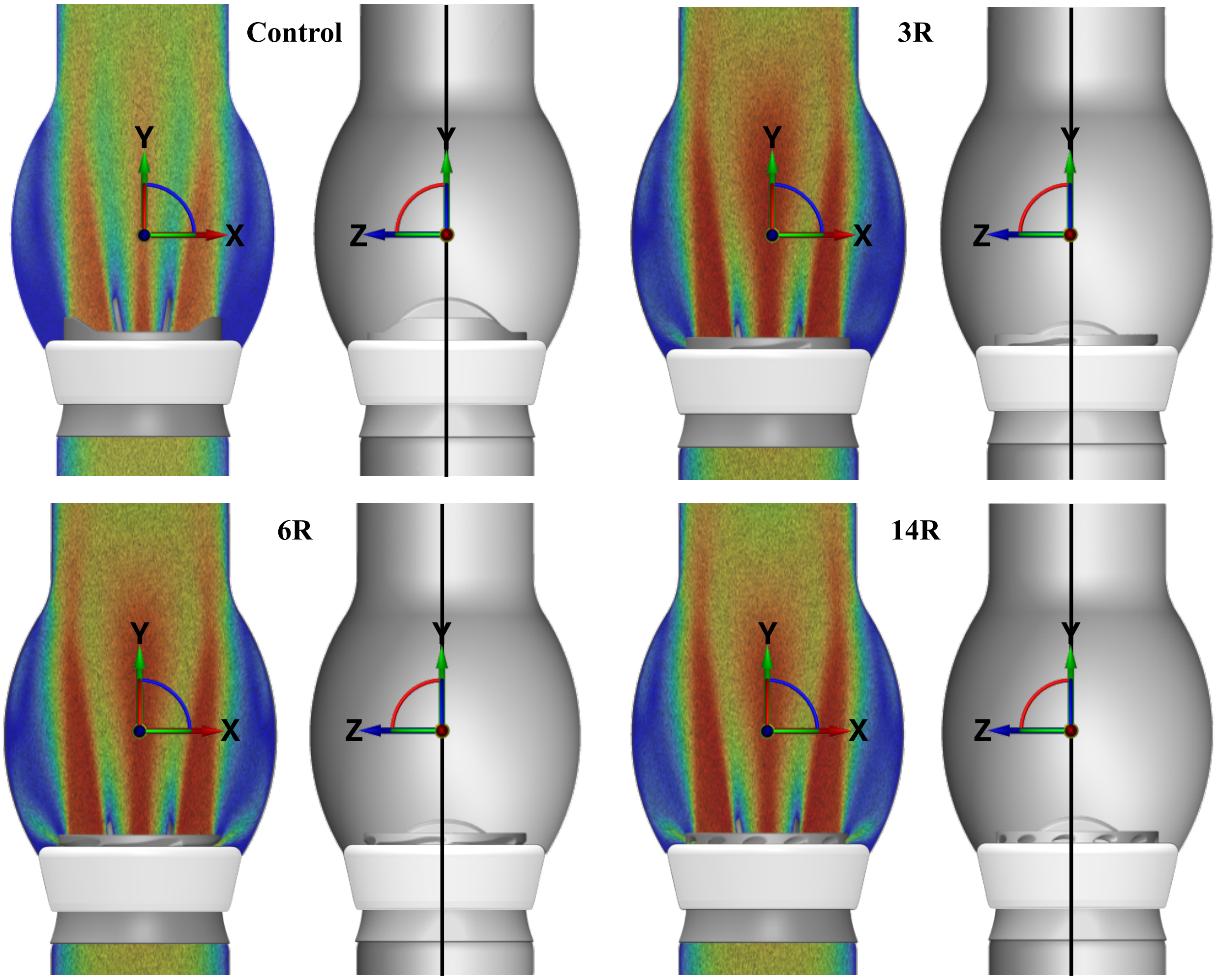

Supplementary Figure S2. Definition of the longitudinal XY plane used to extract the velocity maps shown in Figure 2a. The figure is arranged by valve configuration, with the control valve in the upper-left pair, the 3R valve in the upper-right pair, the 6R valve in the lower-left pair, and the 14R valve in the lower-right pair. For each configuration, the left panel shows the velocity field projected on the XY plane, while the adjacent right panel shows the corresponding geometry viewed normal to this plane. In the orthogonal view, the XY plane is represented by a solid black line, indicating its exact position relative to the aortic root, valve housing, and leaflet region. The coordinate system is shown in each panel for reference, with Y denoting the axial flow direction, X the in-plane radial direction, and Z the direction normal to the extraction plane. This figure demonstrates that all velocity maps in Figure 2a were extracted from the same longitudinal

centerline XY plane, thereby ensuring a consistent comparison of the axial jet structure, sinus flow, and peri-ring velocity field across the control, 3R, 6R, and 14R configurations.

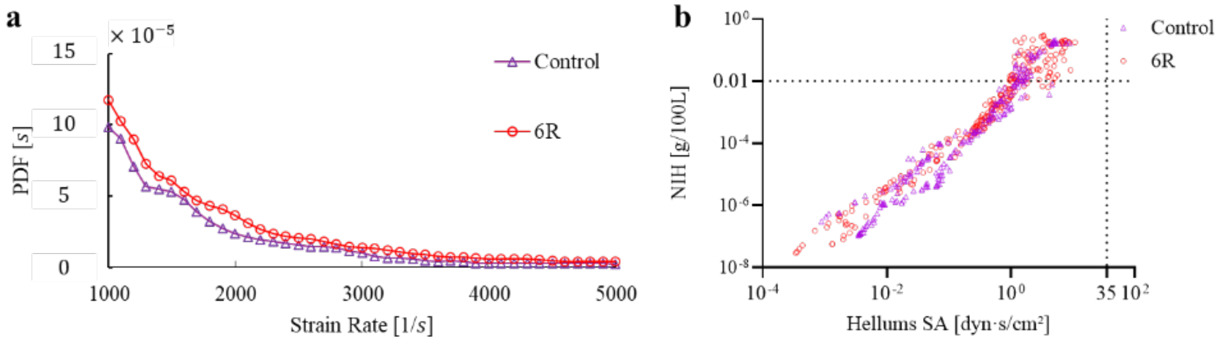

Figure S3. High flow sensitivity analysis at 25 L min^{-1} comparing control and 6R mechanical valve designs. (a) High strain rate tail PDF plotted as PDF versus strain rate over 1000-5000 s^{-1}, comparing control and 6R; tail distributions are similar with modest differences near the lower end of the plotted range. (b) Particle wise shear exposure map plotting NIH hemolysis metric versus Hellums SA on log scaled axes, comparing control and 6R models, computed from the same particle seeding count used in the primary retention analysis. Dashed thresholds indicate the adopted criteria for screening: NIH = 0.01 g (100L)$^{-1}$ and Hellums SA = 35 dyn s cm^{-2}. At 25 L min^{-1}, hemolysis screening thresholds are exceeded for a subset of trajectories for both models, whereas platelet stress accumulation remains below the activation criterion for both models. Notably, hemolysis threshold exceedance occurs in both control and routed configurations, while platelet activation criteria remain below threshold, indicating that this behavior reflects a high-flow sensitivity of the valve system rather than a routing-specific effect.

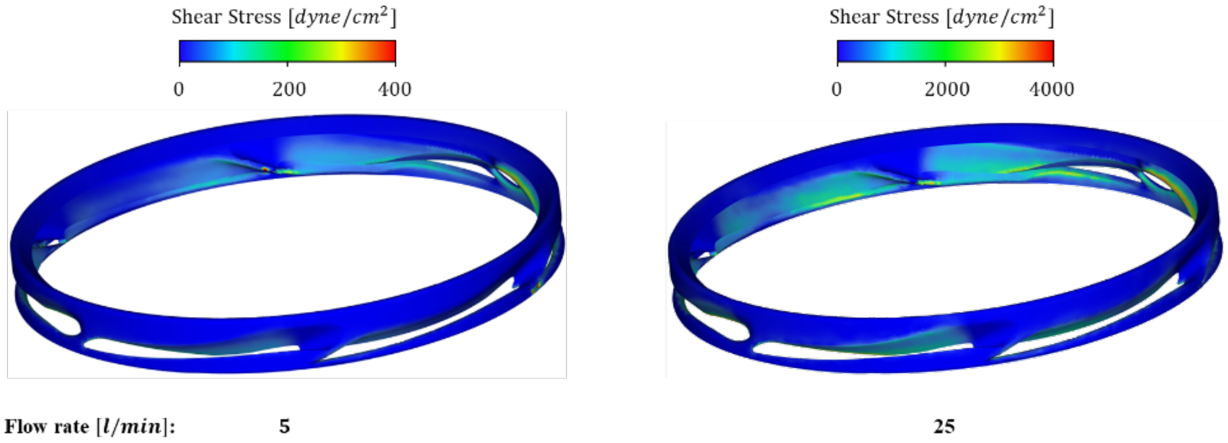

Figure S4. Channel wall shear stress distribution for the 6R routed mechanical valve at 5 and 25 L min⁻¹. Surface maps show wall shear stress magnitude within the routing channel structures. At 5 L min^{-1}, stresses are within the 0 to 400 dyne cm^{-2} range. At 25 L min^{-1}, the distribution shifts upward under 4000 dyne cm^{-2}. These maps address the high shear concern associated with nozzle like routing features under a peak flow sensitivity condition.

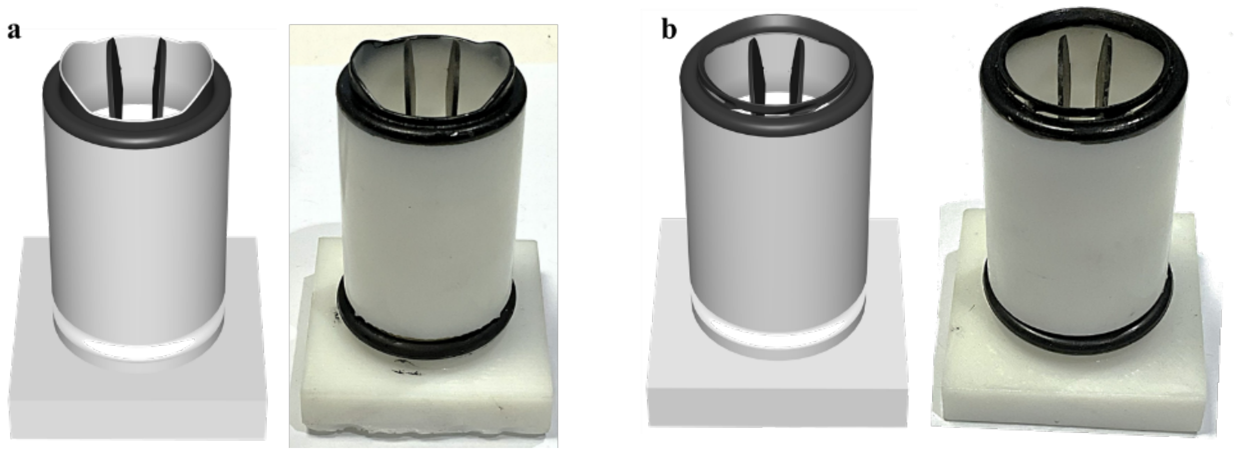

Figure S5. CAD and 3D-printed valve models for in vitro fibrin deposition assays. (a) On the left is a CAD of the control valve on a Pulse Duplicator adapter, with the sewing pad painted black to serve as an optically reactive background for clot deposition. Also on the left is the printed model, produced via high-resolution SLA 3D printing with an ABS-like resin. (b) On the left is a CAD of the 6R routed valve on a Pulse Duplicator adapter, with the sewing pad painted black to serve as an optically reactive background for clot deposition, on which the flow-routing

channels are visible. Also on the left is the printed model, produced via high-resolution SLA 3D printing with an ABS-like resin.

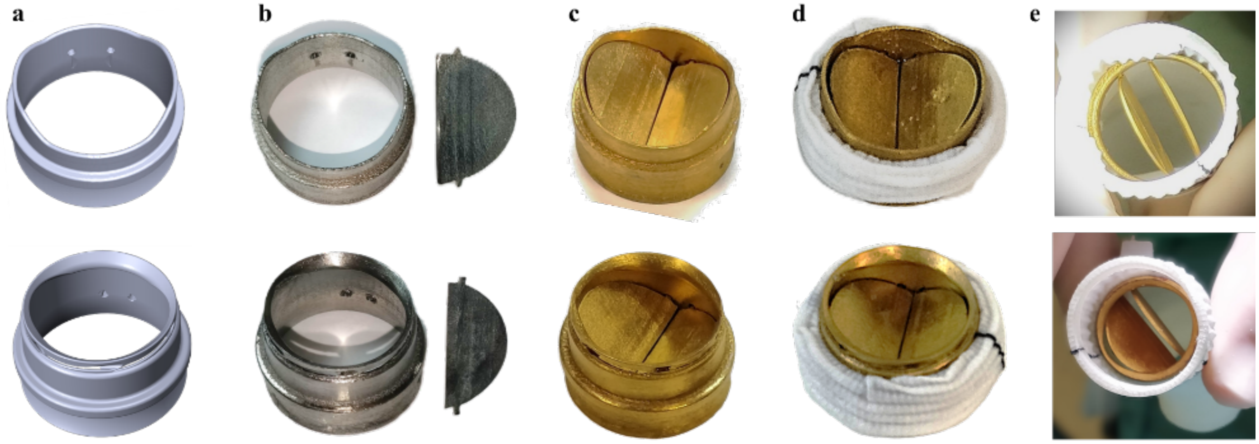

Figure S6. Manufacturing and assembly of titanium valve implants for in vivo evaluation. (a) CAD models of the control and the routed valve housing, top and bottom, respectively. (b) Additive manufacturing process: The 6R routed valve housing was fabricated using Direct Metal Laser Sintering (DMLS) from Ti6Al4V powder. The inset highlights the precise printing of the internal routing channels (bottom). (c) Surface finishing: The printed valves underwent polishing followed by Titanium Nitride (TiN) physical vapor deposition (PVD) coating, resulting in an anticlotting, biocompatible, wear-resistant, gold-colored finish. (d) The valves were inserted into a suture ring and, in their final configuration, prepared for implantation into the native aortic root. (e) Graft integration: The coated valve was sutured into a 24 mm Gelweave Valsalva graft. The photo shows the final implantable construct with the valve seated at the neo-sinus junction.

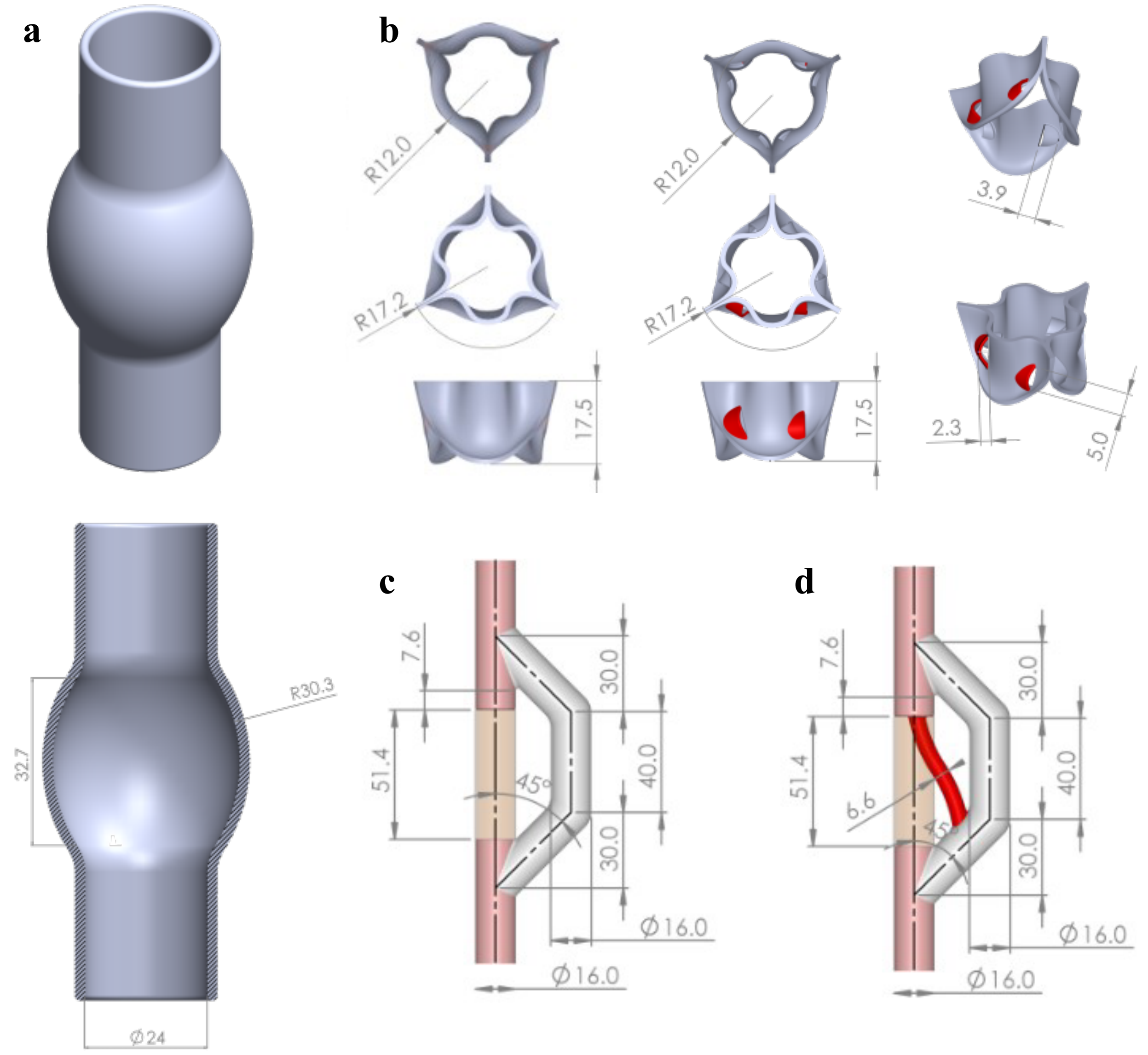

Figure S7. Geometries for polymeric valve and arterial bypass graft extensions. (a) Dimensions of the aorta used for computational verification for all valve models. (b) Dimensions from the bottom view, top view, and side view (upper, middle, and bottom figures respectively) of the polymeric tri-leaflet valve model used for computational verification, showing the leaflet curvature and sinus geometry. (c) Technical specifications for the bottom, top, and side views (upper, middle, and bottom figures, respectively) of the routed polymeric valve variant, along with the dimensions of the routing flaps on the right. (d) Geometry of the arterial bypass graft model, illustrating the anastomotic angle and the blind-ended native artery segment (stump) prone to stagnation. (e) Design of the routed bypass graft that incorporates a pressure-equalizing conduit

(diameter 16.0 mm) connecting the proximal host artery to the stagnant distal segment, to restore flow to the occluded region. All dimensions are in millimeters.

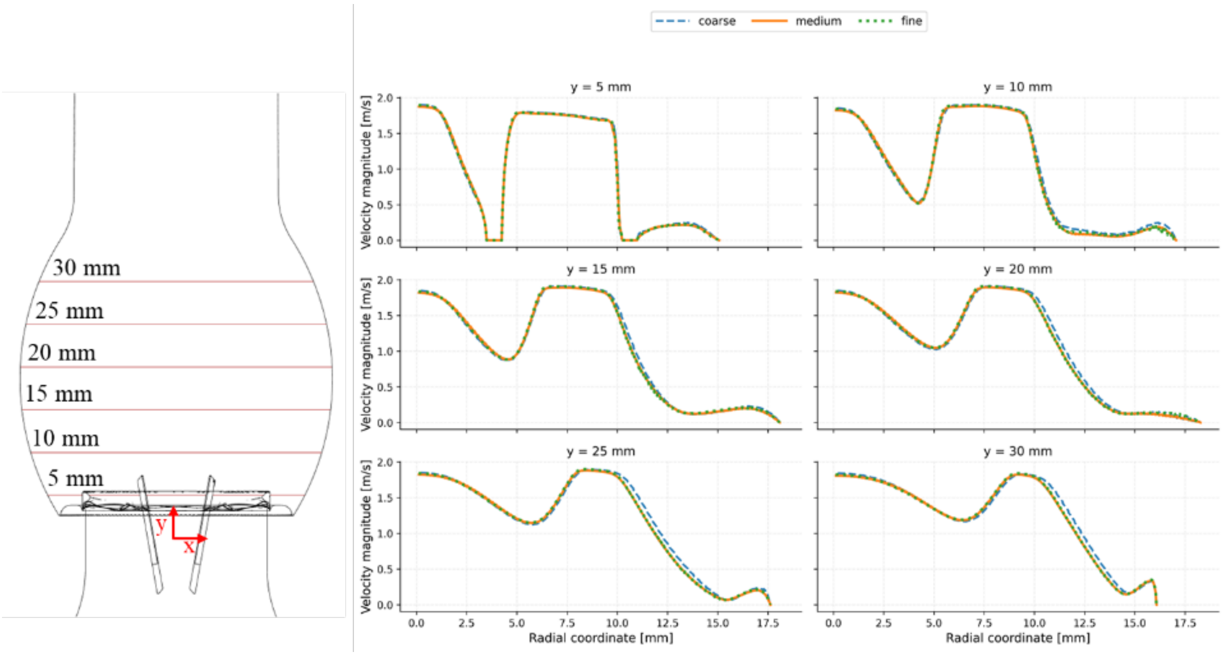

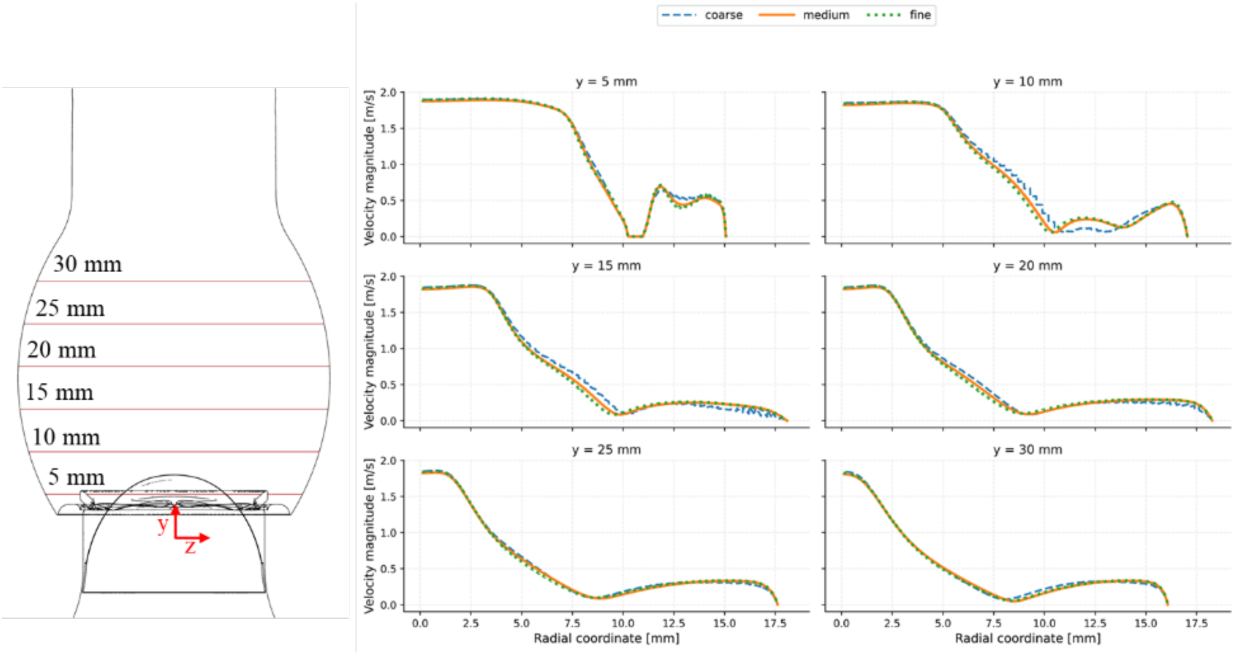

Figure S8. Mesh convergence assessment using velocity-magnitude profiles sampled along radial lines at multiple downstream positions. (a) Velocity magnitude versus radial coordinate

extracted along x-directed radial probe lines at y = 5, 10, 15, 20, 25, and 30 mm downstream of the valve plane (schematic at left). (b) Corresponding profiles extracted along z-directed radial probe lines at the same downstream stations. Three meshes are compared: coarse (2M cells), medium (6M cells), and fine (11M cells). The medium and fine meshes show near-overlap across all stations in both orientations, indicating that the resolved velocity field is largely mesh independent at the fine resolution for the evaluated operating condition. The coarse mesh exhibits larger deviations near steep gradients (jet edges and separation regions), motivating the choice of the fine mesh as the production mesh.

Table S1. CFD mesh details and Lagrangian particle seeding parameters. Summary of the polyhedral mesh cell counts and particle seeding quantities used for residence time quantification and visualization videos across all simulated cases.

CFD Case	Mesh (Number of Cells)	Seeding Amount (Residence Time Analysis)	Seeding Amount (Videos)
**Control MHV**	5M	13,000	250
**3R**	5M	13,000	-
**6R**	5M	21,000	250
**14R**	5M	13,000	-
**Control polymeric valve**	2.5M	-	-
**Routed polymeric valve**	2.5M	-	-
**Control arterial bypass**	400k	-	-
**Routed arterial bypass**	600k	-	-

Legends for Movies

Movie V1: **Time-lapse CFD visualization of particle transport in control and routed (6R) mechanical heart valves.** Time-resolved Lagrangian particle-tracking simulations illustrate the dispersion of inert tracer particles released upstream and advected through the valve domain over a 7-second interval. The control valve (left) displays persistent particle accumulation near the sewing ring and within sinus recirculation regions, while the routed 6R valve (right) shows more continuous particle transport and reduced near-wall retention throughout the simulation.
